# Supplementary material for: Dynamic ROS Control by TIGAR Regulates the Initiation and Progression of Pancreatic Cancer
Source: Cancer Cell. 2020 Feb 10;37(2):168–182.e4. doi: 10.1016/j.ccell.2019.12.012 (PMC7008247; doi:10.1016/j.ccell.2019.12.012)
Supplement: Document S1. Figures S1–S6 [file mmc1.pdf]

Cancer Cell, Volume *37*

Supplemental Information

Dynamic ROS Control by TIGAR Regulates the Initiation and Progression of Pancreatic Cancer

Eric C. Cheung, Gina M. DeNicola, Colin Nixon, Karen Blyth, Christiaan F. Labuschagne, David A. Tuveson, and Karen H. Vousden

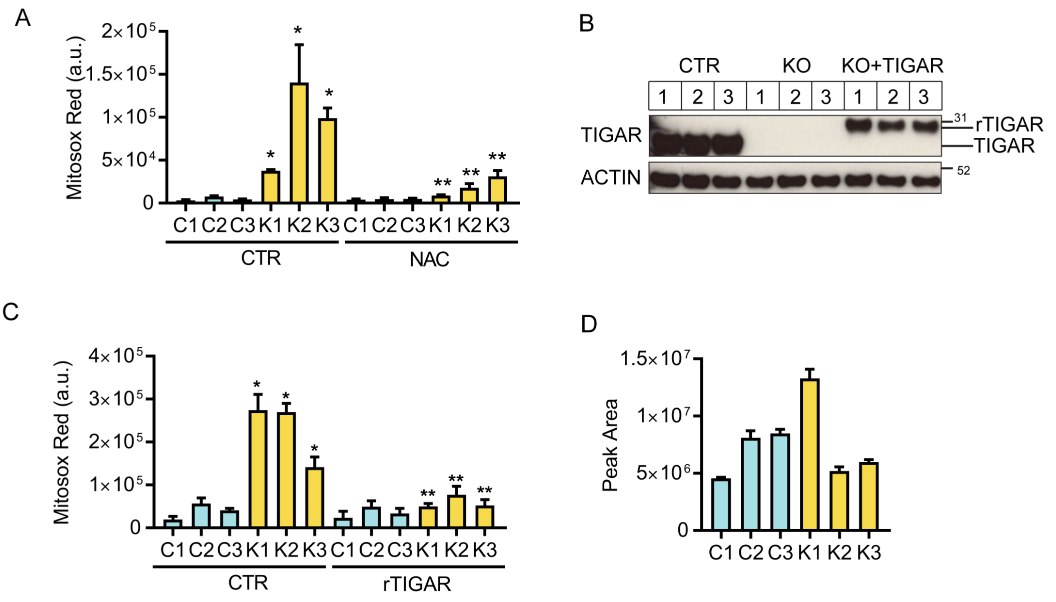

Figure S1. Related to Figure 1. Regulation of mitochondrial ROS by TIGAR (A) Mitochondrial ROS measurement of CTR (C1-3) and TIGAR KO (K1-3) KFC PDAC cells with or without (CTR, no treatment) NAC. * *p<0.05* K1-3 compared to C1-C3, ** *p<0.05* NAC treated K1-3 compared to CTR treated K1-3. (B) Representative Western blot analysis of one set of samples from (C) (CTR, KO and KO+TIGAR KFC PDAC cells) treated with recombinant TIGAR (rTIGAR). ACTIN was used as loading control. (C) Mitochondrial ROS measurement of CTR and KO KFC PDAC cells with or without (CTR, no treatment) rTIGAR. * *p<0.05* K1-3 compared to C1-3, ** *p<0.05* K1-3 with rTIGAR compared to CTR treated K1-3. (D) Mass spectroscopy measurement of ribose 5 phosphate of CTR and KO KFC PDAC cells. (A,C,D) Error bars represent mean ± SEM, and data analysed by one way ANOVA with Tukey post hoc test. n=3 independent experiments for each cell line.

A

KPC

			Histology	
Mouse ID	Sex	Cohort	1°	2°
163208	M	HET	U	
173269	F	HET	G	MD
173271	F	HET	G	C
174844	F	HET	G	
177969	M	HET	G	U
182986	F	HET	G	
183004	M	HET	G	MD
183006	M	WT	U	
183010	F	HET	G	
183014	F	HET	G	
185096	M	HET	G	U
185105	F	HET	G	
186253	M	HET	G	U
186757	F	HET	U	
188464	F	HET	G	U
190411	F	HET	G	
190413	F	HET	G	U
190414	F	HET	G	U
190421	M	WT	G	U
194350	M	HET	G	U
197103	F	WT	G	U
197113	F	HET	G	
197115	F	HET	U	
197412	F	WT	U	
197414	F	WT	G	
199661	M	HET	G	U
199662	M	HET	G	U
199664	M	HET	G	U
200861	F	WT	G	U
203183	M	WT	G	U
200858	M	WT	G	C
185109	F	WT	MD	U
190422	F	WT	MD	U

			Histology	
Mouse ID	Sex	Cohort	1°	2°
163230	F	KO	G	MD
173265	M	KO	G	MD
173921	M	KO	G	U
174843	F	KO	G	MD
178357	F	KO	G	U
178631	F	KO	U	
182052	M	KO	G	MD
182059	F	KO	G	
182990	M	KO	G	
182991	M	KO	G	U
182997	F	KO	G	MD
183869	F	KO	G	MD
183870	F	KO	G	C
186259	F	KO	G	
186746	M	KO	G	
186748	M	KO	G	C
187160	F	KO	G	
187162	M	KO	G	
187163	M	KO	G	
187167	M	KO	G	C
187168	F	KO	G	U
187169	F	KO	G	C
187170	F	KO	G	U
188467	F	KO	G	
190404	M	KO	G	
190407	F	KO	G	U
190972	F	KO	G	
193646	F	KO	G	
193662	M	KO	G	
193663	M	KO	G	
196868	F	KO	G	
198041	M	KO	G	
202762	M	KO	G	U

B

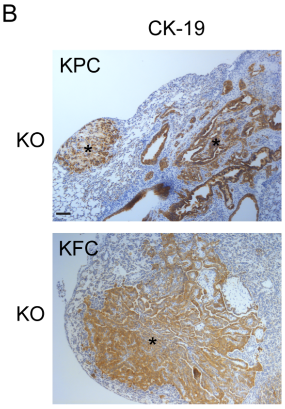

C

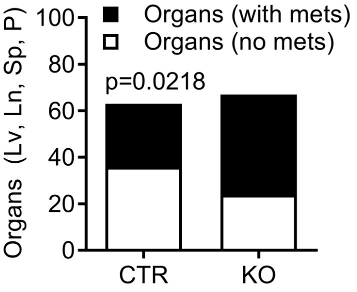

KFC

			Histology	
Mouse ID	Sex	Cohort	1°	2°
OECF15.1f	F	HET	S	U
OECF13.1a	F	HET	U	S
OECF13.1g	F	HET	Norm	G
OECF14.1f	M	HET	Norm	G
OECF20.1c	M	HET	MD	S
OECF15.3c	F	HET	MD	Norm
OECF19.2a	F	HET	Norm	G
OECF19.1d	F	HET	S	G
OECF21.1b	F	HET	G	MD
OECF17.2c	F	HET	U	S
OECF14.4d	M	HET	S	C
OECF15.3a	F	HET	G	MD
OECF20.2h	F	HET	G	MD
OECF22.1c	F	HET	MD	Norm
OECF20.3d	F	HET	G	MD
OECF14.6c	F	WT	Norm	G
OECF28.4f	M	HET	C	U
OECF22.2c	M	HET	G	MD
OECF20.4d	M	HET	U	MD
OECF20.4f	M	HET	C	C
OECF31.1a	F	WT	Norm	MD
OECF32.1c	M	WT	U	
OECF32.1f	M	WT	U	
OECF32.1g	M	WT	U	G
OECF19.5c	M	HET	U	S
OECF19.5f	M	WT	U	S
OECF32.2b	M	WT	MD	S
OECF29.5g	F	WT	G	S
OECF29.6c	M	WT	G	MD
OECF36.1d	M	WT	S	G
OECF36.2b	M	WT	U	S
OECF36.3f	F	WT	MD	S

			Histology	
Mouse ID	Sex	Cohort	1°	2°
OECF16.1b	M	KO	S	U
OECF16.1f	M	KO	G	S
OECF16.1g	M	KO	U	S
OECF15.1c	F	KO	S	G
OECF13.1d	F	KO	Norm	C
OECF16.2a	M	KO	Norm	G
OECF19.1a	M	KO	G	U
OECF17.1g	F	KO	C	C
OECF15.3f	F	KO	MD	G
OECF15.3g	F	KO	U	S
OECF20.2g	F	KO	MD	S
OECF27.2c	M	KO	MD	G
OECF27.2d	M	KO	MD	S
OECF28.3d	M	KO	Norm	MD
OECF28.3i	F	KO	G	MD
OECF13.2g	M	KO	U	
OECF26.3c	F	KO	U	Norm
OECF20.2e	F	KO	Norm	G
OECF14.1g	M	KO	Norm	G
OECF15.2a	M	KO	Norm	G
OECF19.4g	F	KO	U	Norm
OECF17.4e	F	KO	U	S
OECF17.4f	F	KO	U	MD
OECF27.3e	M	KO	G	Norm
OECF28.5a	M	KO	Norm	MD
OECF19.5g	F	KO	S	C
OECF19.5j	F	KO	U	MD

D

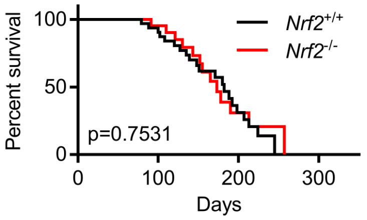

Figure S2. Related to Figure 2. *Tigar* deletion in KRAS driven pancreatic ductal adenocarcinoma (PDAC) (A) Gross pancreatic histology of CTR and KO KPC and KFC mice with PDAC. Norm: normal, G: glandular, MD: moderately differentiated, U: undifferentiated, C:cystic, 1°: greater or equal to 50%

of pancreas, 2°: >25% but <50%. (B) CK-19 staining of lung tissues from *Tigar* KO KPC and KFC. * = PDAC metastasis in lung. Scale bar, 100µm. (C) Numbers of organs (Lv, liver; Ln, lymph node; Sp, spleen; P, peritoneal) of CTR and KO KPC animals with or without metastasis. Data analysed by Fisher exact test. (D) PDAC-free survival of KPC animals (expressing R270H mutant p53) with (Nrf2$^{+/+}$, n = 21) and without NRF2 (Nrf2$^{-/-}$, n = 13). Data were analyzed by log rank test.

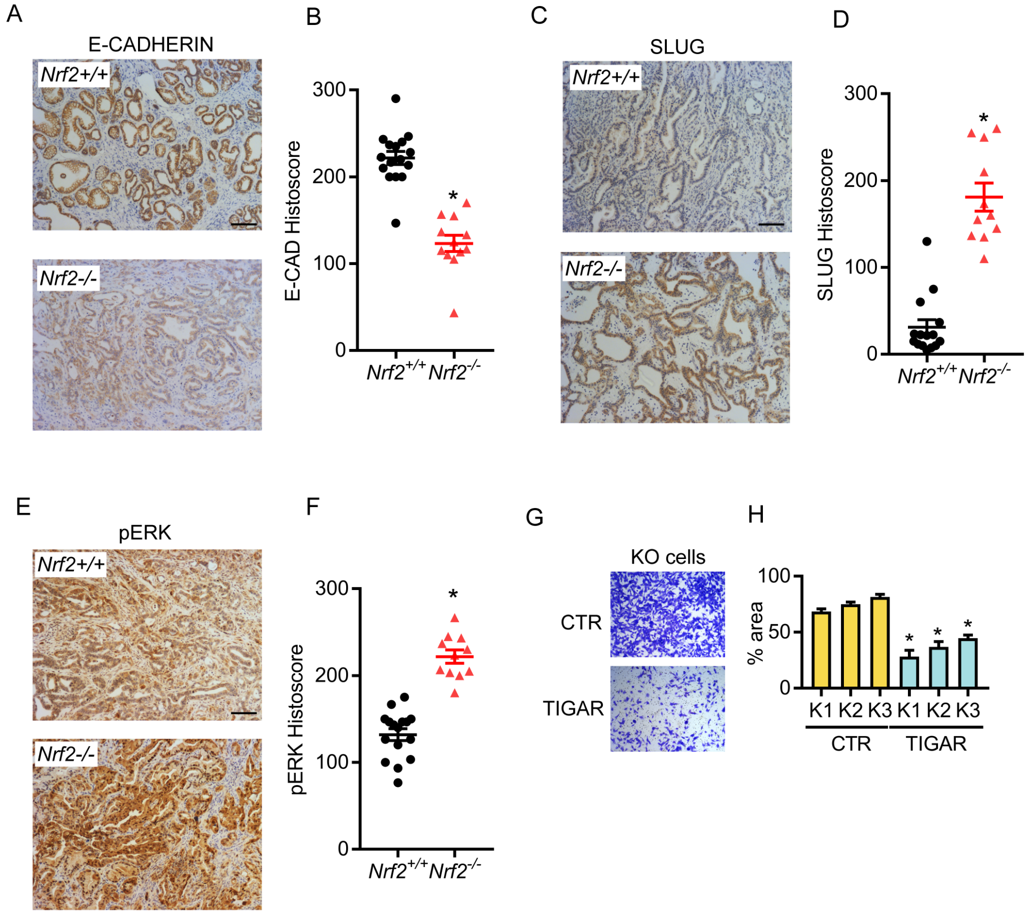

Figure S3. Related to Figure 3. NRF2 loss triggers a ERK-EMT signalling cascade in PDAC, similar to loss of TIGAR. Addition of recombinant TIGAR can decrease the invasiveness of *Tigar* KO PDAC (A and B) E-CADHERIN staining (A) and quantification (B) of *Nrf2*$^{+/+}$ and *Nrf2*$^{-/-}$ KPC tumours. * $p<0.05$ compared to *Nrf2*$^{+/+}$. (C and D) SLUG staining (C) and quantification (D) of *Nrf2*$^{+/+}$ and *Nrf2*$^{-/-}$ KPC tumours. * $p<0.05$ compared to *Nrf2*$^{+/+}$. (E and F) pERK staining (E) and quantification (F) of *Nrf2*$^{+/+}$ and *Nrf2*$^{-/-}$ KPC tumours. * $p<0.05$ compared to *Nrf2*$^{+/+}$. (G and H) Representative images (G) and quantification (H) of transwell migration assay of *Tigar* KO PDAC cells without (CTR) or with (TIGAR) recombinant TIGAR. * $p<0.05$ compared to CTR K1-3. Error bars represent mean ± SEM. (B,D,F) data analysed by two-tailed Student's *t* test, and (H) one way ANOVA with Tukey post hoc test. Scale bar, 100µm.

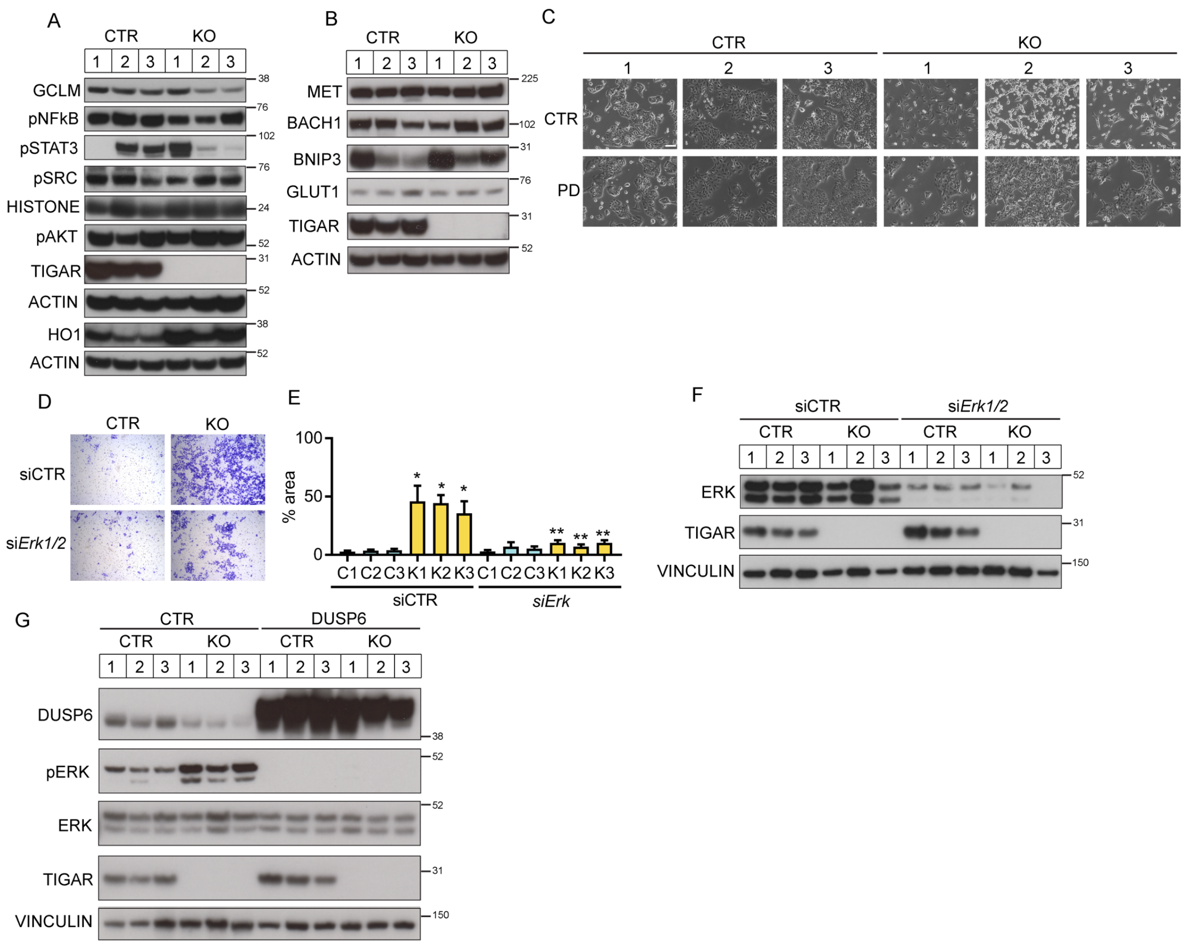

Figure S4. Related to Figure 4. Effect of TIGAR on ROS signalling to drive a mesenchymal phenotype via ERK pathway (A and B) Western blot analysis of CTR and TIGAR KO PDAC KFC cells. (A) GCLM, NFkB, pSTAT3, pSRC with the loading control HISTONE were detected on one blot; pAKT, TIGAR with the loading control ACTIN were detected on a second parallel blot; HO1 with loading control ACTIN (bottom) were detected on a third parallel blot. (B) MET, BACH1, BNIP3, GLUT1, TIGAR with the loading control ACTIN were detected on one blot. (C) Photomicrograph of CTR and TIGAR KO PDAC KFC cells treated without (CTR, vehicle treated) or with PD98059 (PD, 50µM). (D and E) Representative images (D) and quantification (E) of transwell migration assay of CTR and TIGAR KO KFC PDAC cells after knockdown of *Erk*. * *p<0.05* K1-3 compared to C1-3, ** *p<0.05* K1-3 with si*Erk* compared to K1-3 with siCTR. (F) Verification of *Erk* knockdown by Western blot analysis. ERK, TIGAR and the loading control VINCULIN were detected on one blot. (G) Western blot analysis of CTR and TIGAR KO KFC PDAC cells after expression of DUSP6. DUSP6, pERK, ERK, TIGAR and the loading control VINCULIN were detected on one blot. (E) n=3 independent experiments for each cell lines. Error bars represent mean ± SEM, data analysed by one way ANOVA with Tukey post hoc test. Scale bar, 100µm

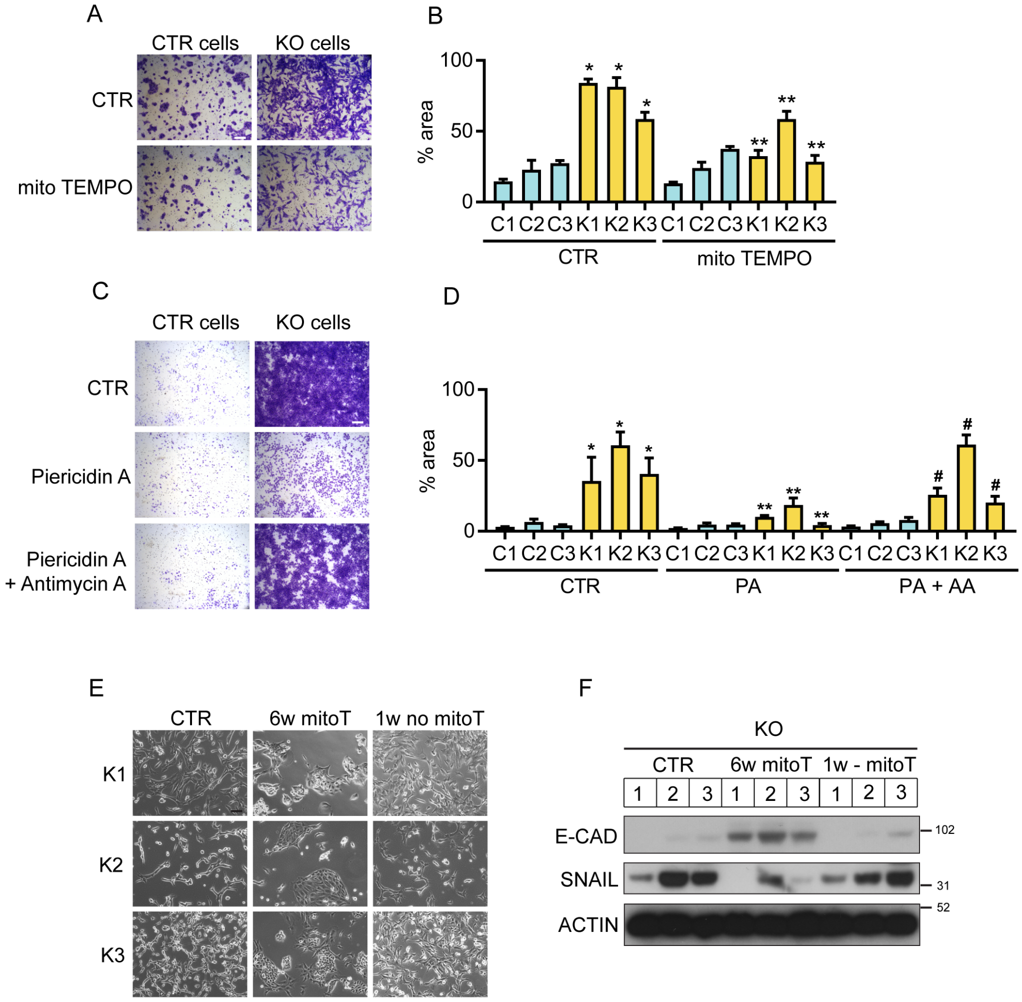

Figure S5: Related to Figure 5. *Tigar* deficiency induced migration can be reduced by limiting mitochondrial ROS. Representative images (A) and quantification (B) of transwell migration assay of CTR and TIGAR KO KFC PDAC cells with mito-TEMPO (100µM) or without (CTR, vehicle). * *p<0.05* K1-3 compared to C1-3, ** *p<0.05* mito-TEMPO treated K1-3 compared to CTR treated K1-3. (C and D) Representative images (C) and quantification (D) of transwell migration assay of CTR and KO PDAC cells with Piericidin-A (PA, 1µM), or combination of Piericidin and Antimycin A (AA, 1µM), or without (CTR, vehicle). * *p<0.05* K1-3 compared to C1-3, ** *p<0.05* PA treated K1-3 compared to CTR treated K1-3, # *p<0.05* PA+AA treated K1-3 compared to PA treated K1-3. (E) Representative images of KO KFC PDAC cells at indicated timepoints continuously treated with mito-TEMPO (mitoT) (50µM) and subsequent removal of mito-TEMPO for one week (1w no mitoT). (F) Representative Western blot analysis of one set of the samples in (E). E-CAD, SNAIL and the loading control ACTIN were detected on one blot. w= week. n=3 independent experiments for each cell lines. (B,D) Error bars represent mean ± SEM, and data analysed by one way ANOVA with Tukey post hoc analysis. Scale bar, 100µm.

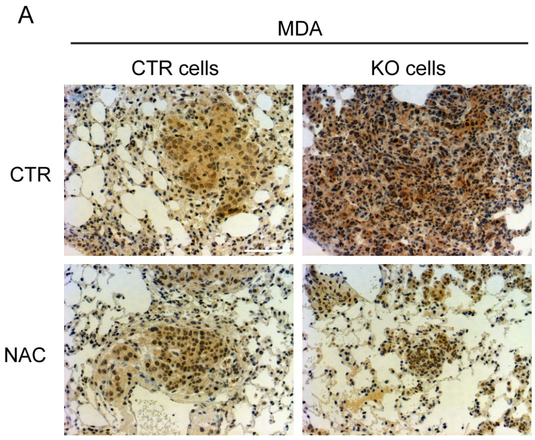

Figure S6. Related to Figure 6. MDA staining in TIGAR deficient metastases. (A) MDA staining of lung tissues from NAC treated or CTR (normal drinking water) treated animals tail vein injected with CTR and KO PDAC KFC cells. Scale bar, 100µm.
