# Supplementary material for: Dynamic ROS Control by TIGAR Regulates the Initiation and Progression of Pancreatic Cancer
Source: Cancer Cell. 2020 Feb 10;37(2):168–182.e4. doi: 10.1016/j.ccell.2019.12.012 (PMC7008247; doi:10.1016/j.ccell.2019.12.012)
Supplement: Document S2. Article plus Supplemental Information [file mmc2.pdf]

Cancer Cell

Article

Dynamic ROS Control by TIGAR Regulates the Initiation and Progression of Pancreatic Cancer

Graphical Abstract

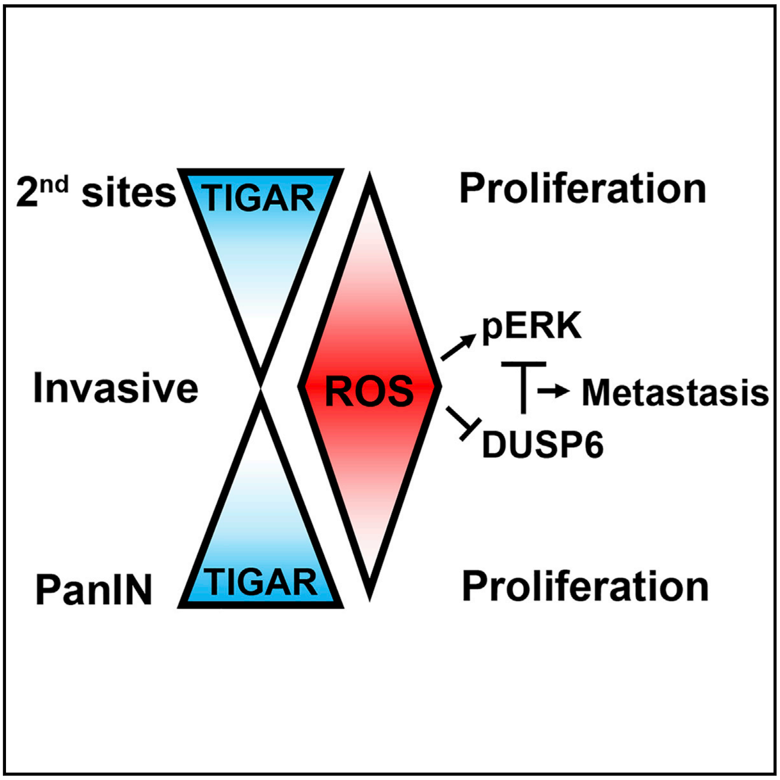

Authors

Eric C. Cheung, Gina M. DeNicola, Colin Nixon, Karen Blyth, Christiaan F. Labuschagne, David A. Tuveson, Karen H. Vousden

Correspondence

karen.vousden@crick.ac.uk

In Brief

Cheung et al. show that TIGAR expression is dynamically regulated during the development of pancreatic ductal adenocarcinoma, resulting in lower levels of ROS to promote tumor initiation in the premalignant condition and higher levels of ROS that enable metastatic progression.

Highlights

- ROS regulation by TIGAR supports premalignant pancreas tumor development
- Increased ROS following TIGAR or Nrf2 loss enhance metastasis
- ROS reduce DUSP6 expression to activate ERK and increase invasion and migration
- TIGAR and ROS levels are dynamically regulated throughout tumor progression

Cheung et al., 2020, Cancer Cell *37*, 168–182
February 10, 2020 © 2020 The Authors. Published by Elsevier Inc.
https://doi.org/10.1016/j.ccell.2019.12.012

CellPress

Cancer Cell
Article

Dynamic ROS Control by TIGAR Regulates the Initiation and Progression of Pancreatic Cancer

Eric C. Cheung,[1] Gina M. DeNicola,[2] Colin Nixon,[3] Karen Blyth,[3,4] Christiaan F. Labuschagne,[1] David A. Tuveson,[5] and Karen H. Vousden[1,6,*]
[1]The Francis Crick Institute, London NW1 1AT, UK
[2]Department of Cancer Physiology, Moffitt Cancer Center and Research Institute, Tampa, FL 33612, USA
[3]Cancer Research UK Beatson Institute, Glasgow G61 1BD, UK
[4]Institute of Cancer Sciences, University of Glasgow, Glasgow G61 1QH, UK
[5]Cold Spring Harbor Laboratory, Cold Spring Harbor, NY 11724, USA
[6]Lead Contact
*Correspondence: karen.vousden@crick.ac.uk
https://doi.org/10.1016/j.ccell.2019.12.012

SUMMARY

The TIGAR protein has antioxidant activity that supports intestinal tissue repair and adenoma development. Using a pancreatic ductal adenocarcinoma (PDAC) model, we show that reactive oxygen species (ROS) regulation by TIGAR supports premalignant tumor initiation while restricting metastasis. Increased ROS in PDAC cells drives a phenotypic switch that increases migration, invasion, and metastatic capacity. This switch is dependent on increased activation of MAPK signaling and can be reverted by antioxidant treatment. In mouse and human, TIGAR expression is modulated during PDAC development, with higher TIGAR levels in premalignant lesions and lower TIGAR levels in metastasizing tumors. Our study indicates that temporal, dynamic control of ROS underpins full malignant progression and helps to rationalize conflicting reports of pro- and anti-tumor effects of antioxidant treatment.

INTRODUCTION

Reactive oxygen species (ROS) play important and diverse roles in regulating many aspects of cell behavior, from signaling proliferation and survival to promotion of oxidative damage and cell death. Not surprisingly, therefore, control of ROS production and defects in antioxidant defense have been associated with many aspects of human health and disease (Schieber and Chandel, 2014). The contribution of ROS to cancer development has been somewhat controversial and is clearly highly complex. Numerous lines of evidence support the concept that ROS contribute to cancer initiation and development. ROS can induce both genotoxic damage and chronic inflammation (Feig et al., 1994; Tafani et al., 2016), while membrane-associated ROS generated through NADPH oxidases (such as NOX4) are important contributors to the activation of signaling pathways that drive proliferation and metastasis (Saikolappan et al., 2019). Furthermore, mitochondrial ROS were also shown to be necessary for KRAS-induced cancer development (Weinberg et al., 2010). It was therefore surprising that no beneficial effect of antioxidants was detected in cancer prevention studies, some of which even revealed an increase in cancer development in the antioxidant-treated group (Alpha-Tocopherol and Beta Carotene Cancer Prevention Study Group, 1994; Klein et al., 2011). Subsequent work has shown that many of the steps involved in malignant progression are associated with increased oxidative stress and an increased sensitivity of cells to succumb to ROS-induced death (Gorrini et al., 2013). Activation of antioxidant defense pathways is critical for successful tumor development, leading to a revised model in which ROS limitation may function to enhance tumorigenesis. Many studies have now supported the concept that tumor cells inherently carry a high

Significance

Many studies and clinical trials have shown ROS to both enhance and retard tumor progression. This study seeks to clarify the effects of ROS over the full course of development of a single tumor type, pancreatic ductal adenocarcinoma. In this model we show that ROS limitation by the antioxidant protein TIGAR is important for the development of premalignancies, while ROS promotion can drive increased metastasis through activation of ERK. We also show that the effects of ROS on tumor invasiveness are reversible, allowing for the dynamic switching from a proliferative to an invasive phenotype, and back. This work rationalizes the complexity of ROS regulation during cancer progression and may help to guide the use of antioxidants in cancer therapy.

 © 2020 The Authors. Published by Elsevier Inc.
This is an open access article under the CC BY license (http://creativecommons.org/licenses/by/4.0/).

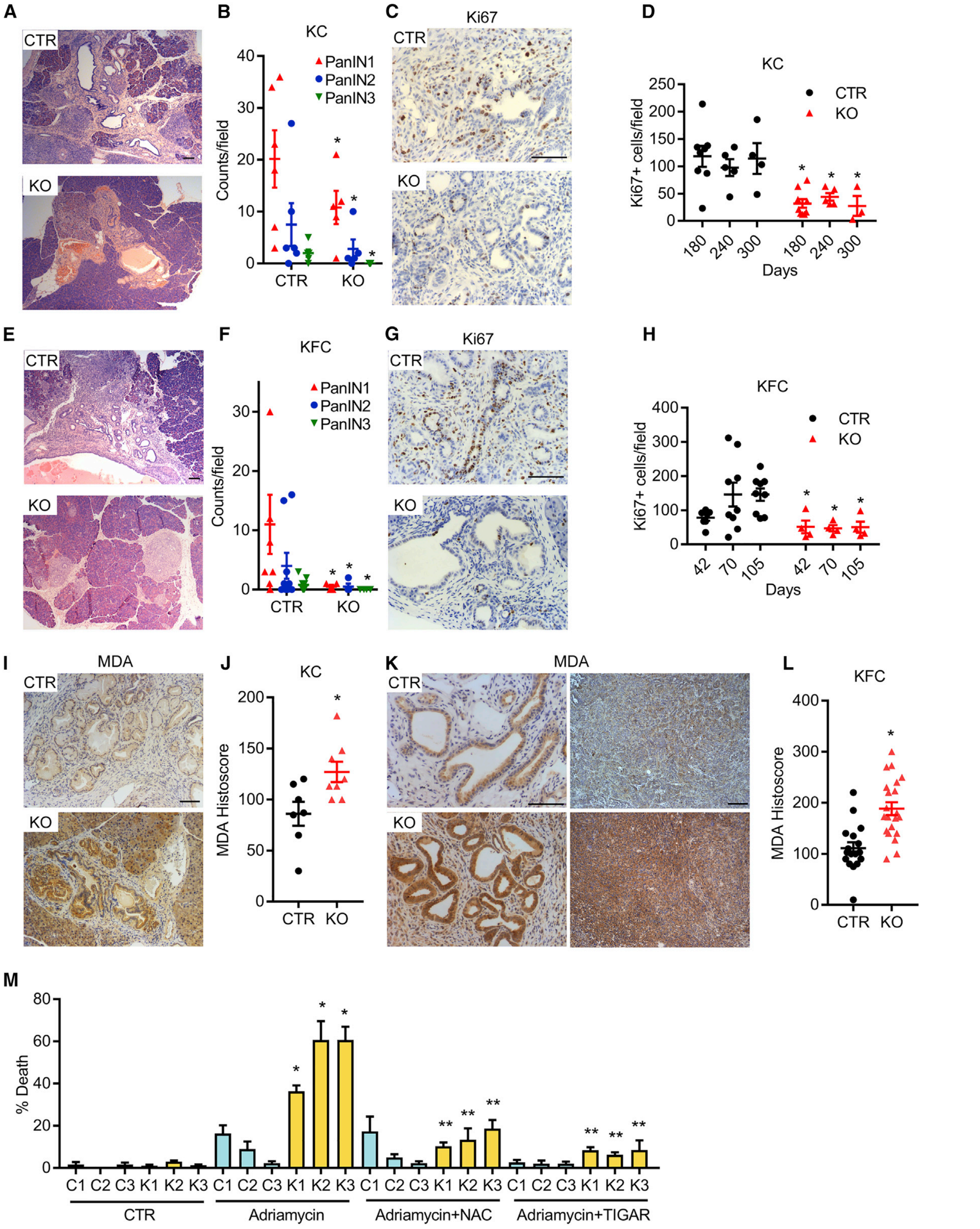

Figure 1. *Tigar* Deletion Reduces Proliferation and PanIN-Precursor Lesions in KRAS-Driven Ductal Adenocarcinoma (PDAC) and Reduces Cell Survival after Oxidative Stress *In Vitro*

(A and B) H&E staining of pancreas lesions (A) and quantification (B) of PanIN from control (CTR) and *Tigar*-deficient (KO) KC mice (CTR, *Pdx1-Cre;LSL-Kras*$^{G12D/+}$*;Tigar*$^{+/+}$ or *Tigar*$^{fl/+}$ [n = 6]; KO, *Pdx1-Cre;LSL-Kras*$^{G12D/+}$*;Tigar*$^{fl/fl}$ [n = 5]) at 240 days. *p < 0.05 compared with CTR.

(C and D) Ki67 staining at 240 days (C) and number of Ki67-positive cells at indicated ages (D) of CTR and KO KC pancreas. *p < 0.05 compared with CTR.

(legend continued on next page)

burden of oxidative stress, reflecting abnormal oncogenic signaling and loss of normal environment. Survival of these cells depends on a concomitant increase in ROS scavenging pathways that is not needed in normal cells, suggesting that interference with these antioxidant pathways or simply additional ROS burden may selectively kill cancer cells. Interestingly, many commonly used chemotherapeutic agents effectively induce ROS (Chandel and Tuveson, 2014; Gorrini et al., 2013).

Many mechanisms through which tumor cells limit ROS exposure have been described. One of the most ubiquitous is the activation of NRF2, a transcription factor that regulates a program of genes involved in antioxidant defense. Activation of oncogenes such as KRAS or environmental signals such as lack of oxygen (hypoxia) can induce NRF2, with evidence that this response is necessary for tumor development (DeNicola et al., 2011; Mitsuishi et al., 2012). Direct activation of NRF2 through overexpression of the protein or loss of the negative regulator KEAP1 is frequent in cancer development (Rojo de la Vega et al., 2018). Another protein that contributes to ROS limitation is TIGAR, a protein with bisphosphatase activity that can support the activation of the oxidative pentose phosphate pathway (PPP) in response to oxidative stress and so enhance the production of NADPH for antioxidant defense (Bensaad et al., 2006; Cheung et al., 2013). Functions of TIGAR that limit ROS have been shown to contribute to damage resolution in intestinal epithelium and protect from pathologies such as cerebral ischemia (Cheung et al., 2013; Li et al., 2014). However, elevated expression of TIGAR has also been detected in many cancer types, consistent with a role for antioxidants in tumor progression (Lee et al., 2014). In mice, loss of TIGAR leads to increased survival and slower tumor development in intestinal and lymphoma models (Cheung et al., 2013; Maddocks et al., 2017).

While ROS limitation has a clear role in supporting cancer development, the contribution of ROS to different stages of cancer development remains unclear. Early studies suggested that increased ROS may be linked to enhanced metastasis (Arnandis et al., 2018; Arora et al., 2013; Goh et al., 2011; Ishikawa et al., 2008; O'Leary et al., 2015; Porporato et al., 2014; Radisky et al., 2005). However, several recent studies in melanoma and other tumor types have elegantly demonstrated an importance of ROS limitation in allowing metastatic spread (Le Gal et al., 2015; Lignitto et al., 2019; Piskounova et al., 2015; Sayin et al., 2014; Wiel et al., 2019). These observations are consistent with studies showing that detachment of cells from the extracellular matrix (Debnath et al., 2002), as may occur when cells leave the primary tumor site and enter the circulation, results in increased ROS, which is limited by various metabolic adaptations. These recent data have led to the suggestion that ROS are required for early stages but suppress later stages of cancer development (Assi, 2017), a model supported by studies in melanoma (Bagati et al., 2019; Le Gal et al., 2015; Piskounova et al., 2015).

In this study, we examine the role of TIGAR in the development of pancreatic cancer. Using mutant KRAS-driven pancreatic ductal adenocarcinoma (PDAC) mouse models, we show that loss of TIGAR delays the emergence of premalignant pancreatic intraepithelial neoplasia (PanIN) lesions, but enhances the metastatic capacity of the tumor cells, leading to decreased survival. *Tigar* null tumors and cells show higher ROS levels and increased mesenchymal characteristics, accompanied by enhanced capacity for migration and invasion. The responses to loss of TIGAR are plastic and reverted by treatment of cells with antioxidants. Consistently, the pattern of TIGAR expression in both human and mouse PDACs also suggests a role for ROS limitation in the establishment of the primary malignancy and distant metastasis, with a role for enhanced ROS during the process of metastatic spread.

RESULTS

Tigar Deletion in KRAS-Driven Pancreatic Cancer Increases ROS and Limits Early Tumor Progression

To examine the role of TIGAR in the development of PDAC, we utilized well-established mouse models that use *Pdx1-Cre* to drive pancreas expression of mutant KRAS (*LSL-Kras*$^{G12D/+}$) alone (KC) or mutant KRAS with mutant p53 (*LSL-p53*$^{R172H/+}$; KPC) or with loss of p53 (*p53*$^{fl/+}$; KFC). In the KFC model, CRE-mediated deletion of one p53 allele is accompanied by loss of the remaining wild-type allele during tumor development (Hingorani et al., 2005). Each of these models was crossed into a *Tigar*$^{fl/fl}$ strain to generate pancreatic tumors that retained Tigar expression (CTR) or deleted (KO) for *Tigar*. Initial analysis of preneoplastic PanIN in the KC model showed that loss of TIGAR delayed the appearance of each stage of PanIN progression (PanIN1, 2, and 3), accompanied by lower proliferation in the *Tigar* null lesions, measured by Ki67 staining (Figures 1A–1D). Using the KFC model, PanIN lesions were detected more rapidly, and again, the loss of TIGAR retarded the appearance of PanIN and lowered proliferation of these preneoplastic lesions (Figures 1E–1H). These results are consistent with our work showing that loss of TIGAR delayed the appearance of intestinal adenomas in response to APC loss and previous work showing decreased PanIN development following loss of the antioxidant factor NRF2 in a PDAC model (Cheung et al., 2013; DeNicola et al., 2011). Using anti-malondialdehyde (MDA) staining of peroxidized lipids as a marker of oxidative stress, we confirmed an increase of ROS in the *Tigar* KO PanINs (in the KC and KFC

(E and F) H&E staining of pancreas lesions (E) and quantification (F) of PanIN from CTR and KO KFC (CTR, *Pdx1-Cre;LSL-Kras*$^{G12D/+}$*;Trp53*$^{fl/+}$*;Tigar*$^{+/+}$ or *Tigar*$^{fl/+}$ [n = 9]; KO, *Pdx1-Cre;LSL-Kras*$^{G12D/+}$*;Trp53*$^{fl/+}$*;Tigar*$^{fl/fl}$ [n = 4]) mice at 70 days. $^{*}p < 0.05$ compared with CTR.

(G and H) Ki67 staining at 70 days (G) and number of Ki67-positive cells at indicated ages (H) of CTR and KO KFC pancreas. $^{*}p < 0.05$ compared with CTR.

(I and J) MDA staining (I) and quantification (J) of CTR and KO KC pancreas at 240 days. $^{*}p < 0.05$ compared with CTR.

(K and L) MDA staining (K) and quantification (L) of CTR and KO KFC pancreas at endpoint. $^{*}p < 0.05$ compared with CTR.

(M) Cell death of CTR (C1–3) and TIGAR KO (K1–3) KFC PDAC cell lines 24 h after Adriamycin (1 μg/mL) treatment alone or with either NAC (1 mM) or recombinant TIGAR (5 μg/mL). $^{*}p < 0.05$ Adriamycin-treated K1–3 compared with Adriamycin-treated C1–3, $^{**}p < 0.05$ compared with Adriamycin-treated K1–3. n = 3 independent experiments for each cell line.

Error bars represent mean ± SEM. Data analyzed by unpaired Student's t test between CTR and KO (B, D, F, H, J, and L) or one-way ANOVA with Tukey post hoc test (M). Scale bar, 100 μm. See also Figure S1.

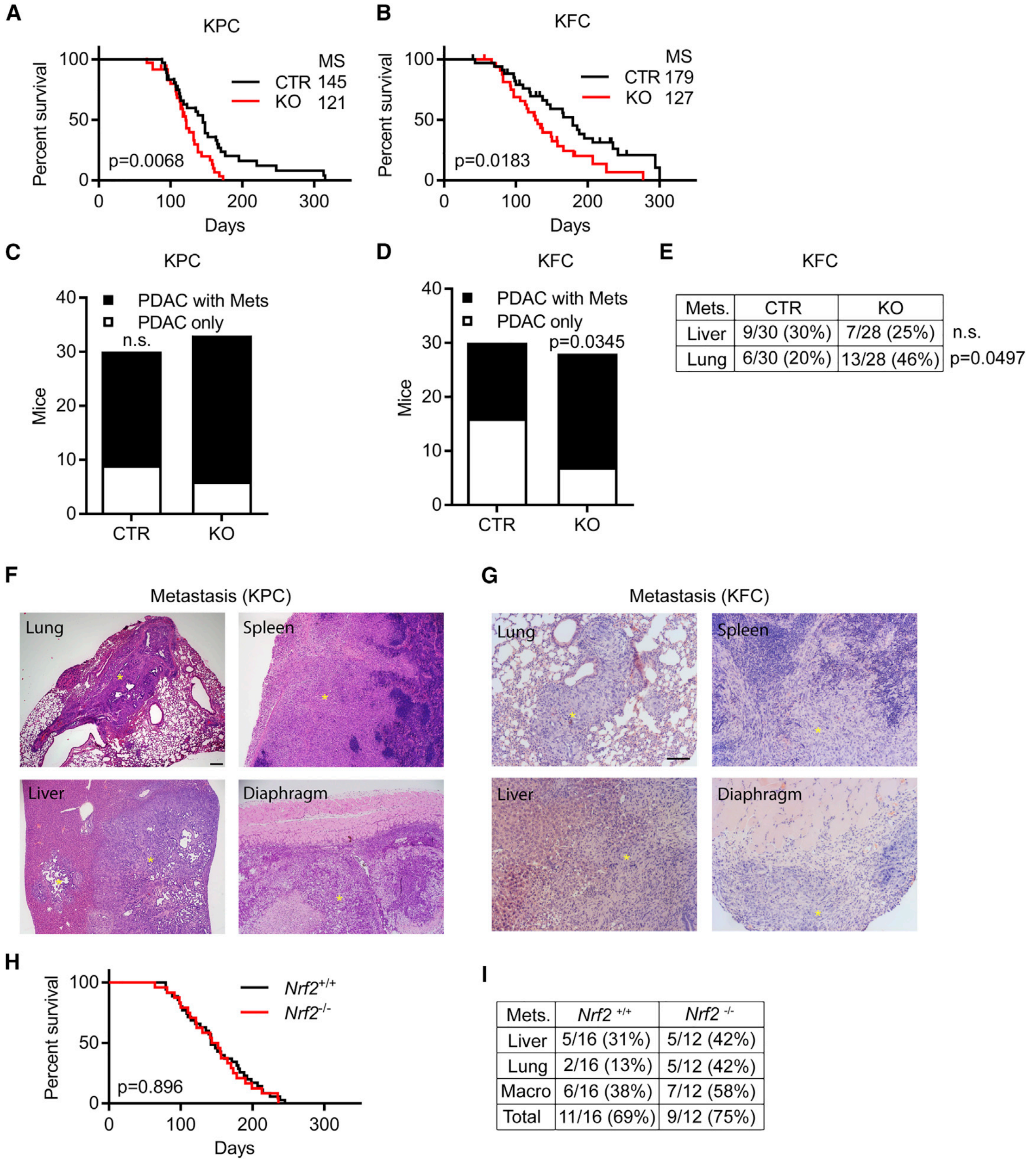

Figure 2. *Tigar* or *Nrf2* Deletion Promotes Invasion and Metastasis in KRAS-Driven Pancreatic Ductal Adenocarcinoma

(A) Disease-free survival in mutant p53-driven PDAC (KPC) with and without TIGAR (CTR, *Pdx1-Cre;Kras*$^{G12D/+}$*;Trp53*$^{R172H/+}$*;Tigar*$^{+/+}$ or *Tigar*$^{fl/+}$ [n = 30]; KO, *Pdx1-Cre;Kras*$^{G12D/+}$*;Trp53*$^{R172H/+}$*;Tigar*$^{fl/fl}$ [n = 28]). MS, median survival in days.

(B) Disease-free survival in p53-deficient-driven PDAC (KFC) with and without TIGAR (CTR, *Pdx1-Cre;Kras*$^{G12D/+}$*;Trp53*$^{fl/+}$*;Tigar*$^{+/+}$ or *Tigar*$^{fl/+}$ [n = 35]; KO, *Pdx1-Cre;Kras*$^{G12D/+}$*;Trp53*$^{fl/+}$*;Tigar*$^{fl/fl}$ [n = 33]). MS, median survival in days.

(C) Numbers of CTR and KO KPC animals with and without metastasis.

(D) Numbers of CTR and KO KFC animals with and without metastasis.

(E) Number of lung and liver metastases in *Tigar* control (CTR) and *Tigar*-deficient (KO) KFC animals.

(F) H&E staining of *Tigar*-deficient KPC tissues with metastasis (denoted by asterisks). Scale bar, 100 μm.

(G) H&E staining of *Tigar*-deficient KFC tissues with metastasis (denoted by asterisks). Scale bar, 100 μm.

(H) Overall survival of KPC animals (expressing R270H mutant p53) with (*Nrf2*$^{+/+}$, n = 34) and without NRF2 (*Nrf2*$^{-/-}$, n = 25).

(I) The number of lung and/or liver metastases in NRF2 wild-type (*Nrf2*$^{+/+}$) and NRF2-deficient (*Nrf2*$^{-/-}$) KPC animals (16 of 21 NRF2 wild-type and 12 of 13 NRF2-deficient mice with PDAC (Figure S2D) were examined). Liver = number of mice with macroscopic metastases in liver; lung = number of mice with macroscopic metastases in lung; macro = number of mice with macroscopic metastases (lung and/or liver); total = mice with macroscopic and/or microscopic metastases in lung and/or liver.

Data in (A), (B), and (H) were analyzed by log rank test. Data in (C)–(E) were analyzed by Fisher's exact test. See also Figure S2.

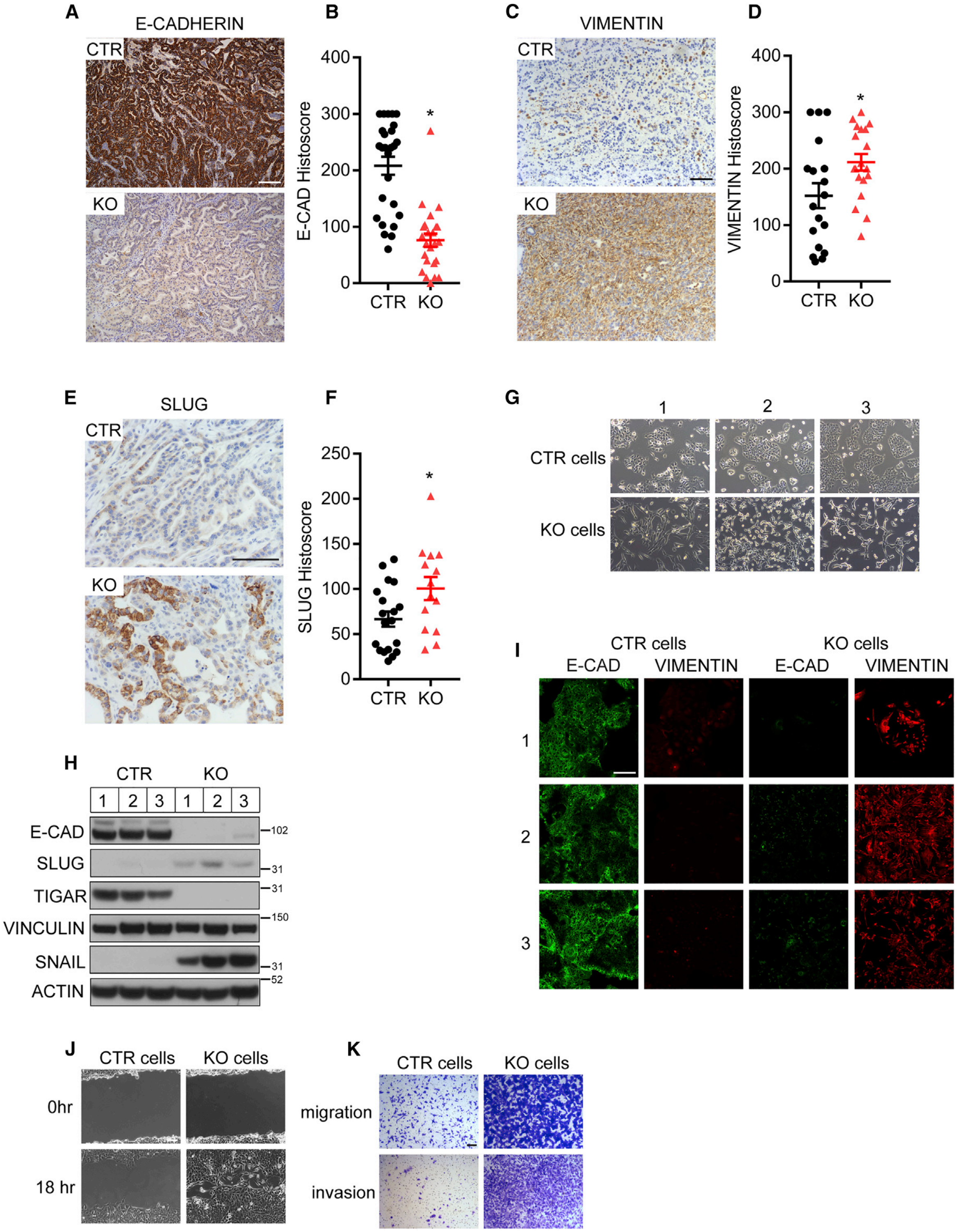

Figure 3. *Tigar* Deletion Promotes Epithelial to Mesenchymal-like Phenotype in PDAC

(A and B) E-CADHERIN staining (A) and quantification (B) of CTR and TIGAR KO (KO) KFC tumors. *p < 0.05 compared with CTR.

(C and D) VIMENTIN staining (C) and quantification (D) of CTR and KO KFC tumors. *p < 0.05 compared with CTR.

(E and F) SLUG staining (E) and quantification (F) of CTR and KO KFC tumors. *p < 0.05 compared with CTR.

(G) Photomicrographs of isolated PDAC cell lines of CTR (from C1–3) and KO (from K1–3) primary KFC tumors.

(H) Western blot analysis of isolated CTR and KO KFC PDAC cell lines. E-CAD, SLUG, TIGAR, and the loading control VINCULIN were detected on one blot, SNAIL and the loading control ACTIN were detected on a separate parallel blot.

(legend continued on next page)

models) as well as *Tigar* KO PDAC (in the KFC model) (Figures 1I–1L). Cell lines were derived from tumors from three *Tigar* wild-type (C1, C2, C3) and three *Tigar*-deleted (K1, K2, K3) KFC mice. Consistent with an antioxidant role for TIGAR, mitochondrial ROS levels were increased in the *Tigar* KO cell lines and could be lowered by treatment with the antioxidant N-acetyl-L-cysteine (NAC) (Figure S1A). The *Tigar* KO cells also showed increased death following exposure to the ROS-inducing chemotherapeutic Adriamycin (Doxorubicin), which was limited by treatment with NAC (Figure 1M). Importantly, introduction of recombinant TIGAR to the *Tigar* null cells (Figure S1B), which decreased ROS levels in *Tigar* KO cells (Figure S1C), also rescued the sensitivity to Adriamycin (Figure 1M). TIGAR has been shown to support flux through the oxidative PPP, which generates NADPH for antioxidant defense (Li et al., 2014). Both oxidative and non-oxidative PPPs produce ribose 5-phosphate (R5P), and previous studies have shown that these mutant KRAS-expressing PDACs increase R5P generation through the non-oxidative pathway (Ying et al., 2012). Interestingly, no consistent differences in R5P levels were detected between *Tigar* wild-type or null cells (Figure S1D), suggesting that any defect in oxidative PPP in *Tigar* null cells is compensated for by an increase in non-oxidative PPP flux. Taken together, these results show that TIGAR limits oxidative stress, a function that correlates with the ability of TIGAR to support the initial stages of PDAC development.

Tigar Deletion in PDAC Promotes Metastasis and Limits Survival

Whereas tumors in the KC mice progress rather slowly, PDAC development is more rapid in KFC and KPC mice (Hezel et al., 2006; Hruban et al., 2006). Surprisingly, however, despite the delay in PanIN development, loss of TIGAR reduced survival of both KPC and KFC mice (Figures 2A and 2B). This reduced survival was not reflected in any obvious difference in the differentiation status of CTR and KO primary tumors (Figure S2A), but was accompanied by widespread tumor dissemination to multiple organs. Staining for CK-19 confirmed that the lung lesions represented metastatic spread of PDAC (Figure S2B). In the KPC model, where most CTR mice showed evidence of metastases (Hingorani et al., 2005; Hruban et al., 2006), the additional effect of TIGAR loss did not reach significance (Figure 2C). Nevertheless, the total number of organs showing evidence of metastasis was significantly increased by *Tigar* deletion (Figures 2F and S2C). In the less metastatic KFC model, loss of TIGAR resulted in significantly more mice carrying metastases (Figures 2D and 2G), with a striking increase in the number of lung lesions (Figure 2E). In support of these observations, loss of the antioxidant defense protein NRF2 in KPC tumors (which has been shown to reduce PanIN development due to increase of ROS; DeNicola et al., 2011) also failed to extend overall survival (Figure 2H) and PDAC-free survival (Figure S2D) and instead promoted increased lung metastases (Figure 2I). (In this NRF2 KPC model, the mutant p53 allele was *LSL-p53*^{R270H}.) Taken together, our data indicate that increased oxidative stress due to loss of TIGAR in KFC and KPC pancreas cancer models delays initial tumor development but enhances metastatic progression at later stages.

Previous studies have shown that metastatic capacity in PDAC tumors is associated with the acquisition of a more mesenchymal phenotype. While the exact contribution of various drivers of epithelial-mesenchymal transition (EMT) has been a topic of discussion (Aiello et al., 2017; Zheng et al., 2015), more recent data support the importance of a switch from an epithelial phenotype—characterized by E-CADHERIN staining—to a more mesenchymal, VIMENTIN-positive appearance of the cancer cells in driving PDAC metastasis (Krebs et al., 2017). Histological analysis of KFC tumors null for *Tigar* (KO) showed a reduction of E-CADHERIN expression (Figures 3A and 3B) and an increase in VIMENTIN expression (Figures 3C and 3D) compared with KFC tumors that retained TIGAR expression (CTR). This shift in phenotype corresponded with increased expression of the mesenchymal marker SLUG in the KO tumors (Figures 3E and 3F). In support of these observations, loss of the antioxidant protein NRF2 also led to a reduction in E-CADHERIN expression (Figures S3A and S3B) and an increase in SLUG expression (Figures S3C and S3D) in PDAC tumors. In culture, cell lines derived from *Tigar* null KFC tumors also showed a clear shift to a more mesenchymal phenotype (Figure 3G), losing E-CADHERIN expression while gaining expression of SLUG and SNAIL (another marker associated with a mesenchymal phenotype) (Lu and Kang, 2019) (Figure 3H). Immunofluorescence analysis confirmed the switch from E-CADHERIN to VIMENTIN expression in the *Tigar* null PDAC cells (Figure 3I). Consistent with the acquisition of a mesenchymal phenotype and increase in metastasis, *Tigar* null cells showed an increased rate of wound healing in a scratch assay (Figure 3J) and increased migration and invasion in transwell assays (Figure 3K). Reintroduction of exogenous TIGAR in these cells reversed the enhanced invasion (Figures S3G and S3H). These results indicate that TIGAR deficiency allows PDAC cells to increase invasiveness and switch to a more mesenchymal phenotype.

TIGAR Deficiency Promotes Activation of Erk Signaling that Supports the Invasive Phenotype

Previous studies have described numerous mechanisms responsible for the switch to a mesenchymal phenotype in metastatic and therapy-resistant tumor cells, several of which have been shown to be induced by increased ROS (Giannoni et al., 2012; Yang et al., 2014). Analysis of pNF-κB, pSRC, pSTAT3, pAKT, and HO1 expression failed to demonstrate a convincing and consistent upregulation of the NF-κB, SRC, STAT3, AKT, or hypoxia pathway in the *Tigar* KO cells compared with CTR cells (Figure S4A). Furthermore, there were no consistent changes in the expression of proteins encoded by HIF1 target genes such as GLUT1 and BNIP3 (Figure S4B). Previous studies have suggested that depletion of TIGAR results in a decrease in

(I) Immunofluorescence of isolated CTR and KO KFC PDAC cell lines.

(J) Representative images of wound-scratch assay of CTR and KO KFC PDAC cell lines.

(K) Representative images of transwell migration and invasion assays of CTR and KO KFC PDAC cell lines.

(B, D, and F) Error bars represent mean ± SEM, and data were analyzed by two-tailed Student's t test. Scale bar, 100 μm. See also Figure S3.

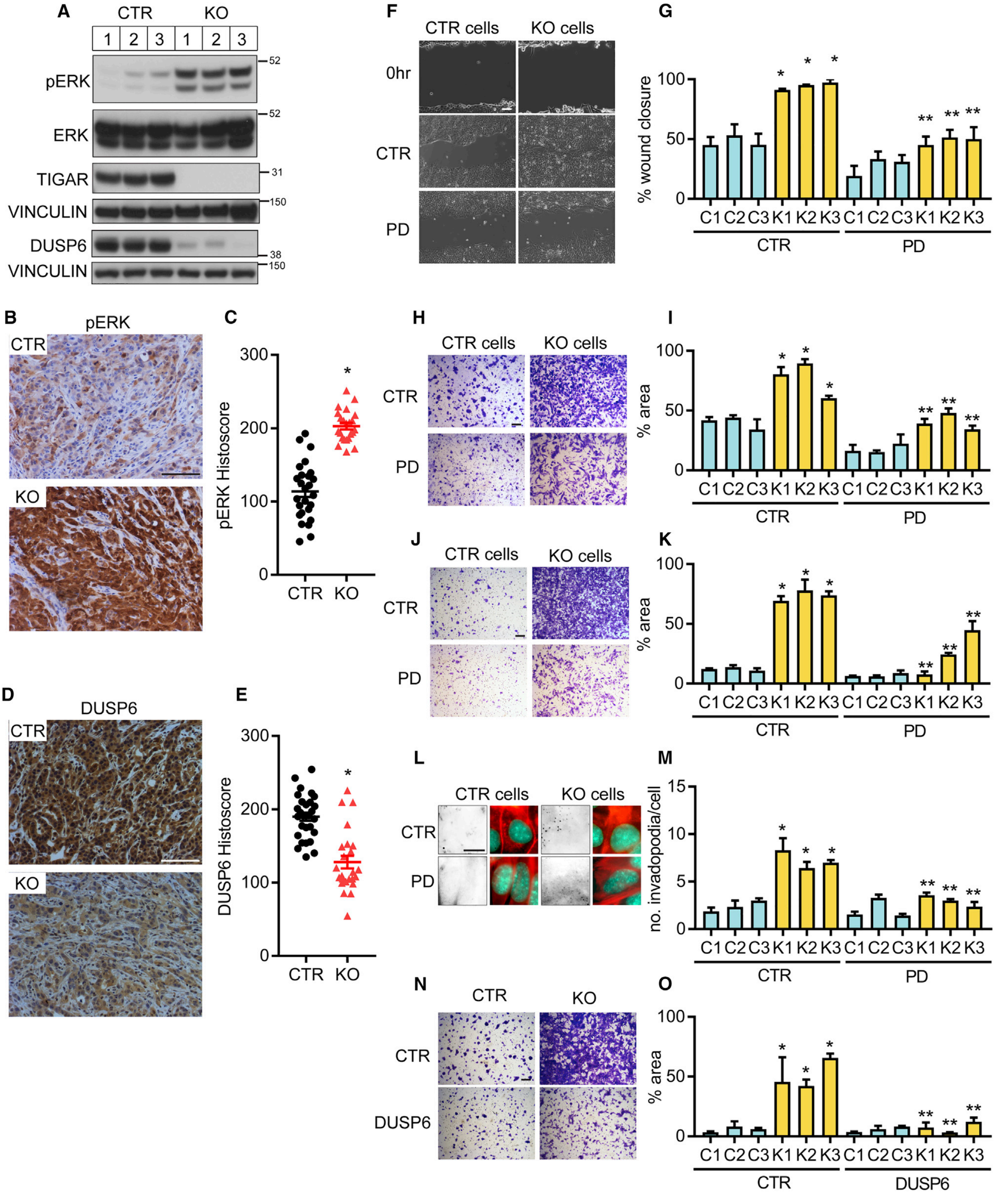

Figure 4. *Tigar* Deficiency Promotes Activation of a Pro-migratory Erk Signaling

(A) Western blot analysis of CTR and TIGAR KO KFC PDAC cells. pERK, ERK, TIGAR, and the loading control VINCULIN were detected on one blot, DUSP6 and the loading control VINCULIN (bottom) were detected on a separate parallel blot.

(B and C) Phospho-ERK (pERK) staining (B) and quantification (C) of CTR and TIGAR KO KFC tumors. *$p < 0.05$ KO compared with CTR.

(D and E) DUSP6 staining (D) and quantification (E) of CTR and TIGAR KO KFC tumors. *$p < 0.05$ KO compared with CTR.

(legend continued on next page)

MET expression in lung cancer cell lines (Shen et al., 2018), but we were unable to detect any difference in MET expression in our PDAC cells (Figure S4B). Recent studies have also shown that increased ROS lead to the degradation of BACH1 to limit metastasis (Wiel et al., 2019), but this was not evident in the TIGAR null PDAC cells (Figure S4B). These data suggest that direct regulation of MET or BACH1 expression does not play a role in the response to loss of TIGAR in our model, although they do not preclude a more general involvement of these signaling pathways. By contrast, a clear increase in phosphorylated ERK was seen in *Tigar* KO cells, accompanied by a decreased expression of DUSP6/MKP-3, the phosphatase responsible for dephosphorylating and inactivating ERK (Groom et al., 1996; Muda et al., 1996) (Figure 4A). This shift in expression correlated with the switch from E-CADHERIN to VIMENTIN expression in these cells (Figure 3I). A similar increase in pERK and decrease in DUSP63 expression was also seen in the PDAC lesions *in vivo* (Figures 4B–4E). Consistently, inhibition of the ERK signaling pathway using the MAPKK inhibitor PD98059 (Pang et al., 1995) led to a reversion of *Tigar* null PDAC cells to a more epithelial phenotype (Figure S4C). Furthermore, a similar increase in phospho-ERK (Figures S3E and S3F) was also observed in NRF2 null PDAC tumors.

To determine the effect of ERK activation on the invasive capacity of *Tigar* KO PDAC cells, we treated the cells with the ERK pathway inhibitor PD98059. The enhanced wound closure, migration, and invasion seen in cells derived from *Tigar* null PDACs were significantly decreased in PD98059 treated cells (Figures 4F–4K). *Tigar* null tumor cells showed an increased collagen-degrading activity indicative of increased invadopodia function (Figures 4L and 4M) that was also dependent on ERK pathway activity. To confirm the contribution of ERK in driving invasion, we showed decreased migration of *Tigar* null PDAC cells depleted of ERK by small interfering RNA (Figures S4D–S4F). Re-expressing DUSP6 in *Tigar* null cells decreased ERK phosphorylation (Figure S4G), as expected, and also decreased the migration of these TIGAR-deficient cells (Figures 4N and 4O). These data are consistent with a model in which *Tigar* null PDAC cells enhance MAPK signaling through a decrease in DUSP6 expression, which drives enhanced migration.

The Role of ROS in TIGAR-Deficiency-Induced Pro-migratory Phenotypes

Having shown increased ROS levels in tumors and cell lines deficient in TIGAR (Figures 1I–1L and S1A) and enhanced sensitivity to oxidative stress for *Tigar* null PDAC cell lines (Figure 1M), we sought to determine the contribution of ROS to the acquisition of the invasive phenotype of these cells. Treatment of *Tigar* KO cells with the antioxidant NAC reduced migration in both wound-scratch and transwell migration assays (Figures 5A–5D), while also limiting invasion (Figures 5E and 5F) and collagen degradation (Figures 5G and 5H). Importantly, the background levels of migration and invasion shown by *Tigar* expressing cells were not significantly decreased by NAC treatment (Figures 5A–5H). This activity in *Tigar* KO cells was also clearly reduced in response to limitation of mitochondrial ROS following treatment with mito-TEMPO (Wipf et al., 2005) (Figures S5A and S5B), suggesting that mitochondrially derived ROS are responsible for the increase in invasiveness in TIGAR-deficient cells. A dose of piercidin that inhibits complex I and so lowers mitochondrially derived ROS also decreased migration in TIGAR-deficient cells (Figures S5C and S5D). Additional treatment with antimycin, which re-establishes mitochondrial ROS production by inhibiting complex III, abrogated this reduction in migration (Figures S5C and S5D), underscoring the importance of mitochondrially derived ROS in the migratory phenotype in TIGAR-deficient cells. Long-term treatment with NAC (Figures 5I and 5J) also reverted the phenotype of the cells, which regained E-CADHERIN and DUSP6 expression and lost SNAIL expression and phosphorylated ERK. TIGAR KO cells also regained E-CADHERIN and lost SNAIL expression after long-term treatment with mito-TEMPO (Figures S5E and S5F). Interestingly, the ROS-dependent control of PDAC morphology and function was highly plastic. *Tigar* KO cells that reverted to epithelial morphology and reduced migration/invasion in response to long-term antioxidant treatment were able to regain these features following removal of the antioxidant (Figures 5I–5L, S5E, and S5F). Taken together, these results show that increased ROS in response to loss of TIGAR can promote a mesenchymal shift accompanied by increased invasive capacity, but that modulating ROS levels can allow the tumor cells to toggle between the two phenotypes.

In Vivo Administration of Antioxidant Can Reduce Lung Metastasis of *Tigar* Null Cells

To test directly whether loss of TIGAR and ROS regulation could have an impact on metastatic capacity, we turned to an experimental model of metastasis in which lung colonization of tumor cells following tail vein injection was assessed. *Tigar* null PDAC cells showed a clearly increased lung colonization

(F and G) Representative images (F) and quantification (G) of wound-scratch assay of CTR (C1–3) and TIGAR KO (K1–3) KFC PDAC cells with or without (CTR, vehicle treatment) MAPK kinase inhibitor PD98059 (PD; 50 μM). *p < 0.05 K1–3 compared with C1–3, **p < 0.05 K1–3 with PD compared with K1–3 without PD (CTR).

(H and I) Representative images (H) and quantification (I) of transwell migration assay of CTR (C1–3) and TIGAR KO (K1–3) KFC PDAC cells with or without (CTR, vehicle treatment) PD98059. *p < 0.05 K1–3 compared with C1–3, **p < 0.05 K1–3 with PD compared with K1–3 without PD (CTR).

(J and K) Representative images (J) and quantification (K) of transwell invasion assay of CTR (C1–3) and TIGAR KO (K1–3) KFC PDAC cells with or without (CTR, vehicle treatment) PD98059. *p < 0.05 K1–3 compared with C1–3, **p < 0.05 K1–3 with PD compared with K1–3 without PD (CTR).

(L and M) Representative images (red, phalloidin; green, nuclei; white, fluorescent gelatin substrate) (L) and quantification (M) of invadopodia assay of CTR and TIGAR KO KFC PDAC cells with or without (CTR, no treatment) PD98059. *p < 0.05 K1–3 compared with C1–3, **p < 0.05 K1–3 with PD compared with K1–3 without PD.

(N and O) Representative images (N) and quantification (O) of transwell migration assay of CTR and TIGAR KO KFC PDAC cells with or without (CTR, empty vector) overexpression of DUSP6. *p < 0.05 K1–3 compared with C1–3, **p < 0.05 K1–3 with overexpressed DUSP6 compared with K1–3 with empty vector.

Error bars represent mean ± SEM. (G, I, K, M, and O) n = 3 independent experiments for each cell line. Data were analyzed by two-tailed Student's t test (C, E) or one-way ANOVA with Tukey post hoc test (G, I, K, M, and O). Scale bar, 100 μm. See also Figure S4.

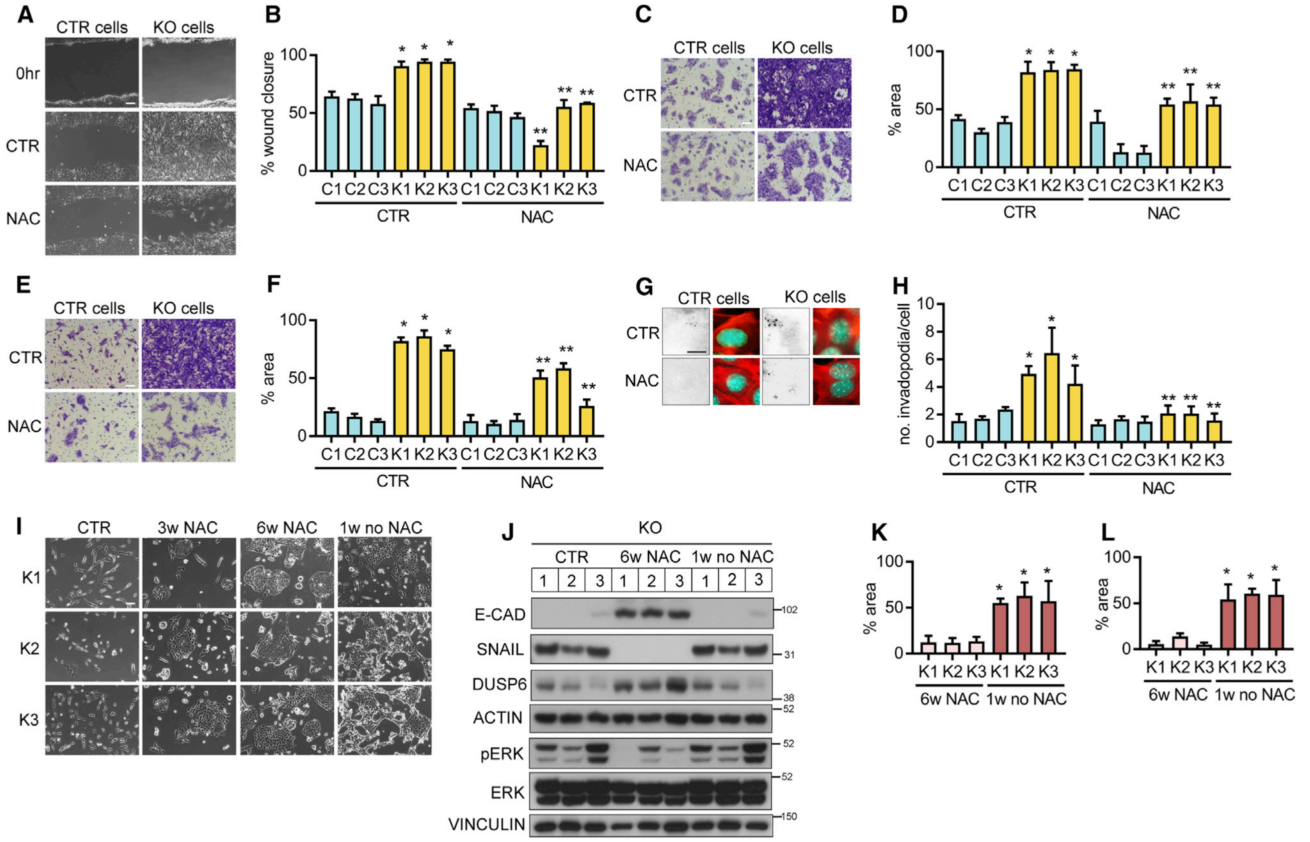

Figure 5. *Tigar*-Deficiency-Induced Pro-migratory Phenotype Can Be Reduced by Antioxidant NAC *In Vitro*

(A and B) Representative images (A) and quantification (B) of *in vitro* wound-scratch assay of CTR (C1–3) and TIGAR KO (K1–3) KFC PDAC cells with NAC (1 mM) or without (CTR, no treatment). *p < 0.05 K1–3 compared with C1–3, **p < 0.05 K1–3 with NAC compared with K1–3 without NAC (CTR).

(C and D) Representative images (C) and quantification (D) of transwell migration assay of CTR (C1–3) and TIGAR KO (K1–3) KFC PDAC cells with NAC (1 mM) or without (CTR, no treatment). *p < 0.05 K1–3 compared with C1–3, **p < 0.05 K1–3 with NAC compared with K1–3 without NAC (CTR).

(E and F) Representative images (E) and quantification (F) of transwell invasion assay of CTR (C1–3) and TIGAR KO (K1–3) KFC PDAC cells with NAC (1 mM) or without (CTR, no treatment). *p < 0.05 K1–3 compared with C1–3, **p < 0.05 K1–3 with NAC compared with K1–3 without NAC (CTR).

(G and H) Representative images (red, phalloidin; green, nuclei; white, fluorescent gelatin substrate) (G) and quantification (H) of invadopodia assay of CTR (C1–3) and TIGAR KO (K1–3) KFC PDAC cells with NAC (1 mM) or without (CTR, no treatment). *p < 0.05 K1–3 compared with C1–3, **p < 0.05 K1–3 with NAC compared with K1–3 without NAC (CTR).

(I) Representative images of TIGAR KO KFC PDAC cells at indicated time points continuously treated with NAC (1 mM) and subsequent removal of NAC for 1 week (1w no NAC).

(J) Western blot analysis of TIGAR KO (1–3) KFC PDAC cells at indicated time points continuously treated with NAC (1 mM) and subsequent removal of NAC for 1 week (1w no NAC). E-CAD, SNAIL, DUSP6, and the loading control ACTIN were detected on one blot; pERK, ERK, and the loading control VINCULIN were detected on a separate parallel blot.

(K) Transwell migration assay of TIGAR KO (K1–3) KFC PDAC cells continuously treated with NAC or with subsequent removal of NAC. *p < 0.05 compared with 6w NAC.

(L) Transwell invasion assay of TIGAR KO (K1–3) KFC PDAC cells continuously treated with NAC or with subsequent removal of NAC. *p < 0.05 compared with 6w NAC.

(B, D, F, H, K, and L) Error bars represent mean ± SEM, n = 3 independent experiments for each cell line, and data were analyzed by one-way ANOVA with Tukey post hoc test. w, weeks. Scale bar, 100 μm. See also Figure S5.

capacity compared with *Tigar* wild-type cells (Figures 6A and 6B), which was decreased following treatment of cells and mice with NAC (Figures 6A and 6B). As expected, *Tigar* KO metastases showed increased ROS (measured by MDA staining) (Figure S6A) that was limited following NAC treatment. Consistent with results in cell lines (Figures 4 and 5), decreased DUSP6 expression and increased phosphorylated ERK were detected in the *Tigar* null lung lesions, a response that was reversed by NAC (Figures 6C–6F). Intriguingly, however, proliferation rates in the *Tigar* KO lung deposits were significantly lower than those seen in the *Tigar* wild-type lesions, a response that was also reversed, to some extent, by NAC treatment (Figures 6G and 6H). While the data are consistent with a ROS-driven increase in the ability of *Tigar* null PDAC cells to colonize the lung following tail vein injection, this decrease in proliferation may indicate that maintenance of high ROS levels becomes detrimental to the proliferation of the lung lesions once the cells have become established at this site.

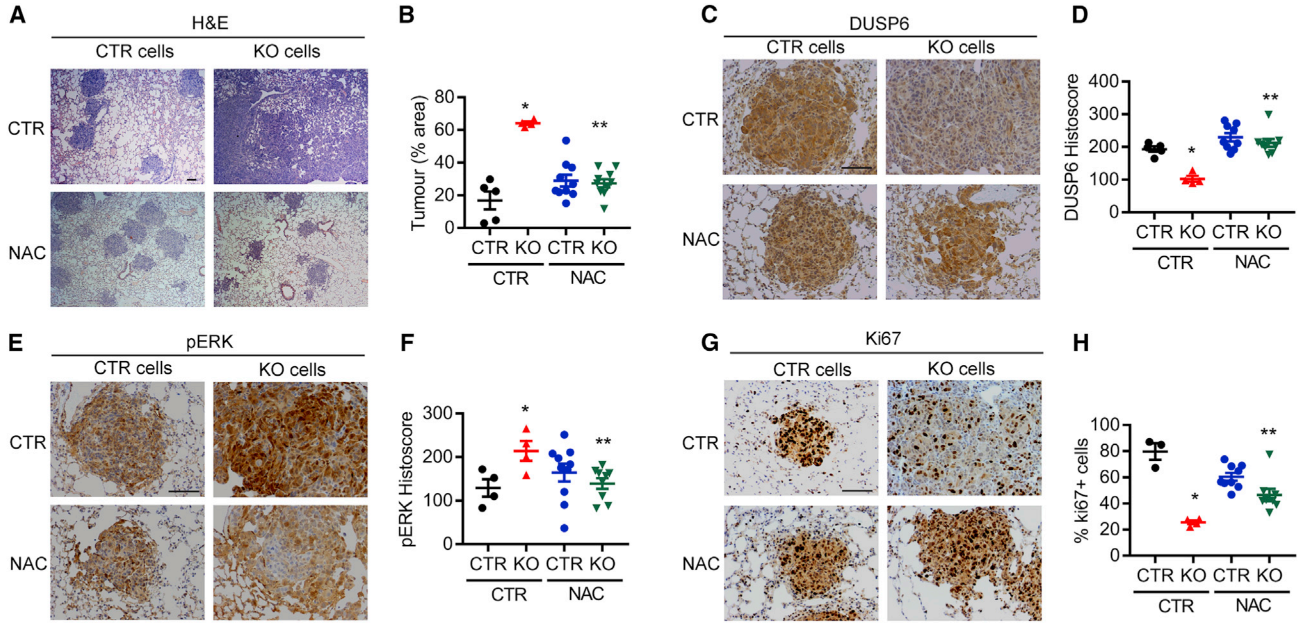

Figure 6. *Tigar*-Deficiency-Induced Metastasis Can Be Decreased by Antioxidant NAC *In Vivo*

Lung tissues from animals 2 weeks after tail vein injection of CTR and TIGAR KO PDAC KFC cell lines with and without NAC treatment (1 g/L drinking water; CTR, drinking water without NAC).

(A and B) (A) H&E staining of lung tissues and (B) quantification of tumor area in lung tissues.

(C and D) DUSP6 staining (C) and quantification (D).

(E and F) pERK staining (E) and quantification (F).

(G and H) Ki67 staining (G) and percentage of Ki67-positive cells (H). (B,D,F,H) *$p < 0.05$ KO compared with CTR, **$p < 0.05$ KO with NAC compared with KO without NAC (CTR).

(B, D, F, and H) Error bars represent mean ± SEM, and data were analyzed by one-way ANOVA with Tukey post hoc test. Scale bar, 100 μm. See also Figure S6.

Dynamic Changes in TIGAR Expression during Cancer Progression

Immunohistochemical analysis of TIGAR at various stages of PDAC tumorigenesis showed an increase in TIGAR expression during the early stages of tumor development in both the KFC mouse model (Figures 7A and 7B) and human PDAC samples (Figures 7C and 7D), consistent with a role for TIGAR in limiting ROS and promoting the survival of these preinvasive cells. However, in both mouse and human cancers, progression to invasive primary tumors was accompanied by a clear decrease in TIGAR expression (Figures 7A–7D), consistent with the selection for cells with higher ROS and higher invasive capacity during these stages of tumorigenesis. Interestingly, TIGAR levels were found to be slightly increased in the metastatic deposits from these tumors, consistent with a role for TIGAR re-expression in limiting ROS and supporting proliferation in the established metastases. Analysis of ROS levels in these different tumor stages showed the expected correlation between high TIGAR and low ROS in PDAC lesions, with higher ROS accumulation in later stage, invasive tumors expressing lower TIGAR levels (Figures 7E and 7F).

DISCUSSION

Many studies in various models have indicated a role for both ROS limitation and ROS promotion in driving tumor initiation and metastatic spread (Arnandis et al., 2018; Ishikawa et al., 2008; Le Gal et al., 2015; Lignitto et al., 2019; O'Leary et al., 2015; Piskounova et al., 2015; Porporato et al., 2014; Radisky et al., 2005; ten Kate et al., 2006; Wiel et al., 2019; Woo et al., 2012). We showed previously that the antioxidant protein TIGAR can support cell survival and proliferation during tissue regeneration and adenoma development, and several studies have shown increased TIGAR expression in various tumor types (Cheung et al., 2013; Lee et al., 2014). Using a pancreatic tumor model, we show that deletion of *Tigar* drives increased ROS and a decrease in the development of premalignant PanIN lesions, consistent with an enhanced proliferative capacity of cancer cells with higher TIGAR expression. However, despite the limitation of tumor initiation, *Tigar* null lesions showed an increased ability to metastasize, especially to the lung. Enhancing ROS through loss of NRF2 in a similar tumor model also limited PanIN development (DeNicola et al., 2011) while promoting enhanced metastasis to the lung. Increased metastatic capacity of the *Tigar* null cells was mirrored by the acquisition of a more mesenchymal phenotype, activation of ERK signaling through the decreased expression of the phosphatase *Dusp6/MKP-3*, and increased migration and invasion *in vitro*. Activation of ERK, while associated with tumor proliferation, migration, and survival, can also limit growth through mechanisms such as senescence or increased sensitivity to other stresses, especially when hyperactivated in the context of an increase of ROS level (Cagnol and Chambard, 2010; Hong et al., 2018;

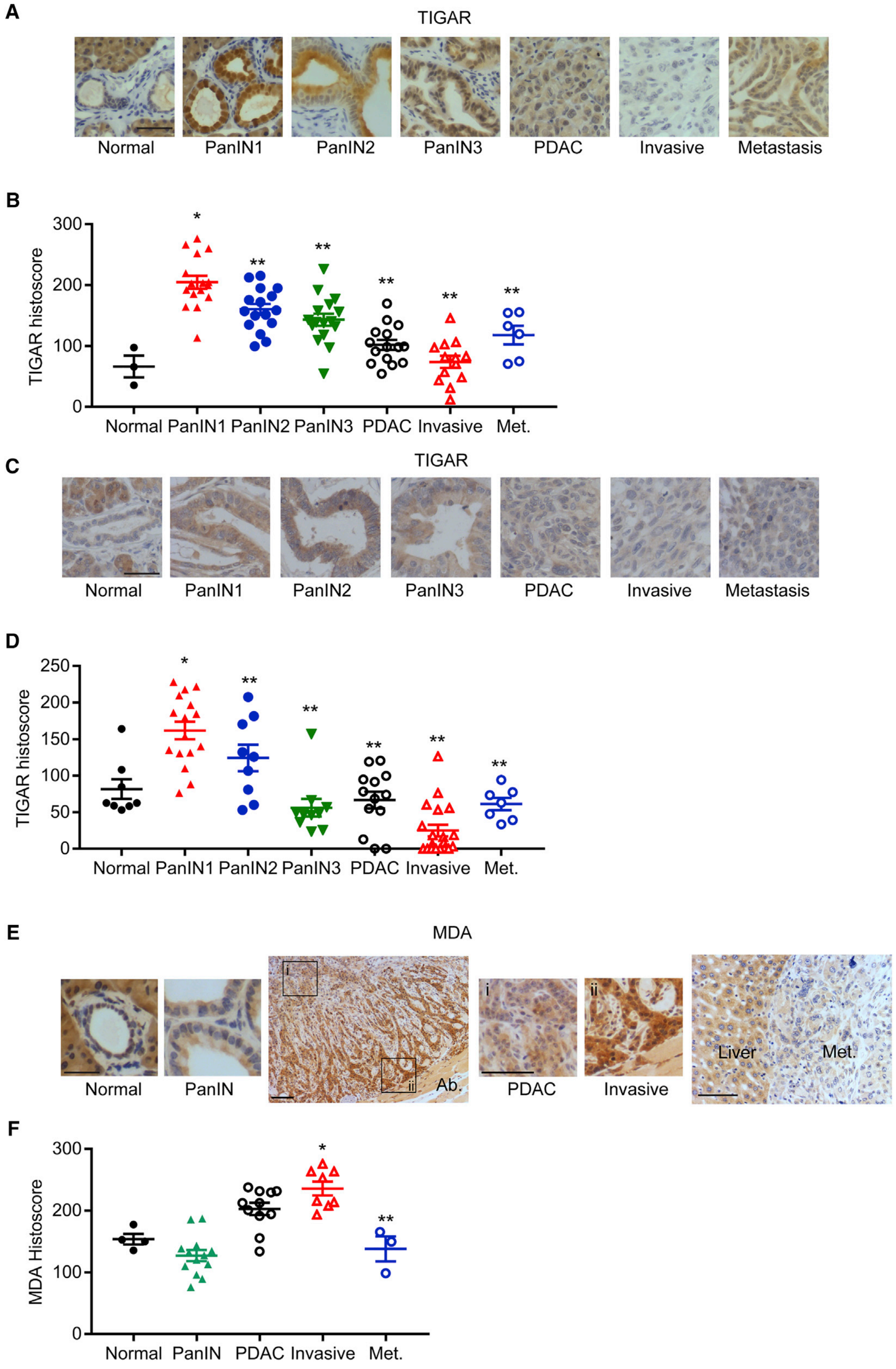

(legend on next page)

Joneson and Bar-Sagi, 1999; Woods et al., 1997). It seems likely that DUSP6 expression can be fine-tuned to modulate ERK at different stages of tumor progression and that this ability is lost in TIGAR null cells. Interestingly, expression of the phosphatase *Dusp6/MKP-3* has been shown previously to be lost in some human PDAC samples, and its expression can be regulated by ROS (Chan et al., 2008; Furukawa et al., 1998). ROS limitation reversed the more aggressive phenotype of these cells and reduced the experimental metastatic capacity *in vivo.*

Our data indicate a complex role for ROS in regulating cancer initiation, growth, and metastatic progression that may help to explain some of the apparently contradictory results seen in previous studies. It is clear that loss of antioxidant defense can limit some stages of tumor development while enhancing others. The exact response of any tumor system is likely to depend on numerous factors, including the cell of origin, the genetic landscape of the tumor cell, and the tumor environment. For example, a failure to limit ROS is clearly detrimental to the metastatic ability of melanoma and lung tumor cells (Le Gal et al., 2015; Lignitto et al., 2019; Piskounova et al., 2015; Wiel et al., 2019), while ROS limitation is associated with enhanced EMT in breast cancer models (Dong et al., 2013). The contribution of different environments in determining the response to ROS is also evident in the increased metastasis seen in both *Tigar* and *Nrf2* deletion models. In cell culture models, the ability of tumor cells to survive matrix detachment—an event that is thought to mirror the success of cells in surviving in the circulation—has also been related to an ability to modulate metabolism to limit ROS (Labuschagne et al., 2019; Schafer et al., 2009; Jiang et al., 2016). However, in the PDAC model described here, enhanced ROS clearly promotes the acquisition of certain mesenchymal phenotypes that can be important in metastasis (Chaffer et al., 2016).

The differential ability of ROS regulation to modulate different steps in the progression of a single tumor type highlights the challenges that would accompany the development of anti- or pro-oxidant approaches for cancer therapy. Although the response to ROS may reflect the overall level of oxidative stress, previous studies have shown that different ROS species, or different locations of ROS production in cells, can have differential effects on proliferation and survival (Goh et al., 2011; Ishikawa et al., 2008; Liou et al., 2016; Schriner et al., 2005). Whether the responses to ROS during tumor progression reflect overall levels of ROS, differences in the cell's ability to respond to ROS or a subtler contribution of different types of ROS that may be controlled by different pathways remain to be determined. We note that in this and previous studies, TIGAR was shown to more effectively limit mitochondrial than cytosolic ROS (Cheung et al., 2016). Whereas in intestinal regeneration studies, this activity clearly functioned to support cell survival and proliferation (and thereby tissue repair), in the context of PDAC progression, the limitation of mitochondrial ROS by TIGAR dampens the activation of signaling pathways that promote migration and invasion.

Our study underscores the multifaceted role of ROS in controlling disease progression. Therapies that either increase or decrease ROS could lead to very different outcomes in different tumor types or at different stages of cancer progression. In intestinal adenomas, which are not metastatic, deletion of TIGAR limits the development of the tumor and improves survival. However, despite the delay in premalignant tumor development in the PDAC model, the capacity of these cells to metastasize is enhanced by TIGAR deletion and decreases overall survival. We speculate that while therapies to increase ROS could be beneficial in some contexts (for example, in lung cancer or melanoma), an initial effect of such treatment on locally confined tumors (such as pancreas) could lead to an inadvertent increase in invasiveness, which is the more likely cause of death. Additional studies to determine the roles of different antioxidants in other genetic mouse models of cancer will be important to inform therapy decisions relating to ROS level manipulation and the timing/sequence of combined treatments involving ROS.

In the PDAC model, ROS can be a powerful regulator of cell phenotype and behavior, allowing cells to toggle between an epithelial/less invasive state to a more mesenchymal/invasive state. The plasticity of this switch is of interest, suggesting that the response is driven by events such as modulation of chromatin modifications that can be reversed when the oxidative signal is removed, and it is important to note that the switch in phenotype requires several weeks in culture in the presence or absence of an antioxidant. A wealth of previous data shows that a switch to a more mesenchymal phenotype can promote successful metastasis (Chaffer et al., 2016). However, the ability to reverse this transition—to undergo mesenchymal-to-epithelial transition—has been linked with the success of cancer cells to expand and proliferate once they have reached and become established at a distant metastatic site (Lu and Kang, 2019; Tsai et al., 2012). The ability of ROS regulation to toggle cells between these two states suggests this could be an important mechanism to regulate many steps of malignant progression. Our analyses of TIGAR expression correlate with a role for ROS limitation in the outgrowth of both the premalignant and the metastatic lesions (selecting for cells with high TIGAR expression), but a contribution of increased ROS in cells during the process of invading or moving to a distant site (selecting for cells with lower TIGAR expression). Our data also hint at a role for a second round of TIGAR upregulation to support proliferation of cells once they are established at the metastatic site. Further studies will be required to understand the mechanisms of TIGAR regulation that can result in this complex pattern of expression.

Figure 7. TIGAR Expression in PDAC and Metastasis

(A and B) TIGAR staining (A) and quantification (B) of mouse KFC PDAC tissues.

(C and D) TIGAR staining (C) and quantification (D) of human PDAC tissue microarray. (B,D) *$p < 0.05$ compared with normal, **$p < 0.05$ compared with PanIN1.

(E and F) MDA staining (E) and quantification (F) of mouse KFC PDAC tissues at various stages of tumor development. Ab., abdominal muscle; Met., metastasis.

(B, D, and F) Error bars represent mean ± SEM, and data were analyzed by one-way ANOVA with Tukey post hoc test. (F) *$p < 0.05$ compared with PDAC, **$p < 0.05$ compared with invasive. Scale bar, 100 μm.

STAR★METHODS

Detailed methods are provided in the online version of this paper and include the following:

- KEY RESOURCES TABLE
- LEAD CONTACT AND MATERIALS AVAILABILITY
- EXPERIMENTAL MODEL AND SUBJECT DETAILS
  - *In Vivo* Animal Studies
  - Transgenic Mouse Models for Pancreatic Ductal Adenocarcinoma (PDAC)
  - Lung Metastasis Model
  - Cell Cultures
- METHOD DETAILS
  - Transwell Migration/Invasion Assays
  - Wound Scratch Assay
  - Histology and Immunohistochemistry
  - Human TMA Analysis
  - Cell Death, ROS Measurement, and Western Blot Analysis
  - Transfection of siRNA and cDNA
  - Immunofluorescence Staining
  - Invadopodia Assay
  - Metabolomics
- QUANTIFICATION AND STATISTICAL ANALYSIS
- DATA AND CODE AVAILABILITY

SUPPLEMENTAL INFORMATION

Supplemental Information can be found online at https://doi.org/10.1016/j.ccell.2019.12.012.

ACKNOWLEDGMENTS

We would like to thank the histology teams and animal facilities at the CRUK Beatson Institute and the Francis Crick Institute. This work was funded by Cancer Research UK grants C596/A10419 and C596/A26855 and supported by the Francis Crick Institute, which receives its core funding from Cancer Research UK (FC001557), the UK Medical Research Council (001557), and the Wellcome Trust (001557), and the CRUK Beatson Institute, which receives its core funding from Cancer Research UK grant C596/A17196. D.A.T. is a distinguished scholar of the Lustgarten Foundation and director of the Lustgarten Foundation-designated Laboratory of Pancreatic Cancer Research. D.A.T. is also supported by the Cold Spring Harbor Laboratory Association, the V Foundation, and the National Institutes of Health (NIH 5P30CA45508, 5P50CA101955, P20CA192996, 1U10CA180944, U01CA224013, U01CA210240-01A1, 1R01CA188134, and 1R01CA190092).

AUTHOR CONTRIBUTIONS

The study was conceived and designed by E.C.C. and K.H.V. Investigations were conducted by E.C.C., G.M.D., C.N., C.F.L., and K.B. The work was supervised by D.A.T., K.B., and K.H.V. The manuscript was written by E.C.C., G.M.D., C.F.L., D.A.T., K.B., and K.H.V.

DECLARATION OF INTERESTS

K.H.V. is on the board of directors and a shareholder of Bristol Myers Squibb, a shareholder of GRAIL, Inc., and on the science advisory board of PMV Pharma, RAZE Therapeutics, Volestra Therapeutics,Inc and Ludwig Cancer. She is a co-founder and consultant of Faeth Therapeutics, funded by Khosla Ventures. She has been in receipt of research funding from Astex Pharmaceuticals and AstraZeneca and contributed to CRUK Cancer Research Technology filing of Patent Application WO/2017/144877. D.A.T. serves on the scientific advisory board and holds shares in Leap Therapeutics and Surface Oncology. D.A.T. serves on the SAB of Cygnal Therapeutics. D.A.T. has research support from ONO Therapeutics, FibroGen, and Halozyme.

Received: June 10, 2019
Revised: October 2, 2019
Accepted: December 21, 2019
Published: January 23, 2020

REFERENCES

Aiello, N.M., Brabletz, T., Kang, Y., Nieto, M.A., Weinberg, R.A., and Stanger, B.Z. (2017). Upholding a role for EMT in pancreatic cancer metastasis. Nature *547*, E7–E8.

Alpha-Tocopherol; Beta Carotene Cancer Prevention Study Group. (1994). The effect of vitamin E and beta carotene on the incidence of lung cancer and other cancers in male smokers. N. Engl. J. Med. *330*, 1029–1035.

Arnandis, T., Monteiro, P., Adams, S.D., Bridgeman, V.L., Rajeeve, V., Gadaleta, E., Marzec, J., Chelala, C., Malanchi, I., Cutillas, P.R., et al. (2018). Oxidative stress in cells with extra centrosomes drives non-cell-autonomous invasion. Dev. Cell *47*, 409–424.e9.

Arora, S., Bhardwaj, A., Singh, S., Srivastava, S.K., McClellan, S., Nirodi, C.S., Piazza, G.A., Grizzle, W.E., Owen, L.B., and Singh, A.P. (2013). An undesired effect of chemotherapy: gemcitabine promotes pancreatic cancer cell invasiveness through reactive oxygen species-dependent, nuclear factor kappaB- and hypoxia-inducible factor 1alpha-mediated up-regulation of CXCR4. J. Biol. Chem. *288*, 21197–21207.

Assi, M. (2017). The differential role of reactive oxygen species in early and late stages of cancer. Am. J. Physiol. Regul. Integr. Comp. Physiol. *313*, R646–R653.

Bagati, A., Moparthy, S., Fink, E.E., Bianchi-Smiraglia, A., Yun, D.H., Kolesnikova, M., Udartseva, O.O., Wolff, D.W., Roll, M.V., Lipchick, B.C., et al. (2019). KLF9-dependent ROS regulate melanoma progression in stage-specific manner. Oncogene *38*, 3585–3597.

Bensaad, K., Tsuruta, A., Selak, M.A., Vidal, M.N., Nakano, K., Bartrons, R., Gottlieb, E., and Vousden, K.H. (2006). TIGAR, a p53-inducible regulator of glycolysis and apoptosis. Cell *126*, 107–120.

Cagnol, S., and Chambard, J.C. (2010). ERK and cell death: mechanisms of ERK-induced cell death–apoptosis, autophagy and senescence. FEBS J. *277*, 2–21.

Chaffer, C.L., San Juan, B.P., Lim, E., and Weinberg, R.A. (2016). EMT, cell plasticity and metastasis. Cancer Metastasis Rev. *35*, 645–654.

Chan, D.W., Liu, V.W., Tsao, G.S., Yao, K.M., Furukawa, T., Chan, K.K., and Ngan, H.Y. (2008). Loss of MKP3 mediated by oxidative stress enhances tumorigenicity and chemoresistance of ovarian cancer cells. Carcinogenesis *29*, 1742–1750.

Chandel, N.S., and Tuveson, D.A. (2014). The promise and perils of antioxidants for cancer patients. N. Engl. J. Med. *371*, 177–178.

Cheung, E.C., Athineos, D., Lee, P., Ridgway, R.A., Lambie, W., Nixon, C., Strathdee, D., Blyth, K., Sansom, O.J., and Vousden, K.H. (2013). TIGAR is required for efficient intestinal regeneration and tumorigenesis. Dev. Cell *25*, 463–477.

Cheung, E.C., Lee, P., Ceteci, F., Nixon, C., Blyth, K., Sansom, O.J., and Vousden, K.H. (2016). Opposing effects of TIGAR- and RAC1-derived ROS on Wnt-driven proliferation in the mouse intestine. Genes Dev. *30*, 52–63.

Debnath, J., Mills, K.R., Collins, N.L., Reginato, M.J., Muthuswamy, S.K., and Brugge, J.S. (2002). The role of apoptosis in creating and maintaining luminal space within normal and oncogene-expressing mammary acini. Cell *111*, 29–40.

DeNicola, G.M., Karreth, F.A., Humpton, T.J., Gopinathan, A., Wei, C., Frese, K., Mangal, D., Yu, K.H., Yeo, C.J., Calhoun, E.S., et al. (2011). Oncogene-induced Nrf2 transcription promotes ROS detoxification and tumorigenesis. Nature *475*, 106–109.

Dong, C., Yuan, T., Wu, Y., Wang, Y., Fan, T.W., Miriyala, S., Lin, Y., Yao, J., Shi, J., Kang, T., et al. (2013). Loss of FBP1 by Snail-mediated repression

provides metabolic advantages in basal-like breast cancer. Cancer Cell *23*, 316–331.

Feig, D.I., Reid, T.M., and Loeb, L.A. (1994). Reactive oxygen species in tumorigenesis. Cancer Res. *54*, 1890s–1894s.

Furukawa, T., Yatsuoka, T., Youssef, E.M., Abe, T., Yokoyama, T., Fukushige, S., Soeda, E., Hoshi, M., Hayashi, Y., Sunamura, M., et al. (1998). Genomic analysis of DUSP6, a dual specificity MAP kinase phosphatase, in pancreatic cancer. Cytogenet. Cell Genet. *82*, 156–159.

Giannoni, E., Parri, M., and Chiarugi, P. (2012). EMT and oxidative stress: a bidirectional interplay affecting tumor malignancy. Antioxid. Redox Signal. *16*, 1248–1263.

Goh, J., Enns, L., Fatemie, S., Hopkins, H., Morton, J., Pettan-Brewer, C., and Ladiges, W. (2011). Mitochondrial targeted catalase suppresses invasive breast cancer in mice. BMC Cancer *11*, 191.

Gorrini, C., Harris, I.S., and Mak, T.W. (2013). Modulation of oxidative stress as an anticancer strategy. Nat. Rev. Drug Discov. *12*, 931–947.

Groom, L.A., Sneddon, A.A., Alessi, D.R., Dowd, S., and Keyse, S.M. (1996). Differential regulation of the MAP, SAP and RK/p38 kinases by Pyst1, a novel cytosolic dual-specificity phosphatase. EMBO J. *15*, 3621–3632.

Hezel, A.F., Kimmelman, A.C., Stanger, B.Z., Bardeesy, N., and Depinho, R.A. (2006). Genetics and biology of pancreatic ductal adenocarcinoma. Genes Dev. *20*, 1218–1249.

Hingorani, S.R., Wang, L., Multani, A.S., Combs, C., Deramaudt, T.B., Hruban, R.H., Rustgi, A.K., Chang, S., and Tuveson, D.A. (2005). Trp53R172H and KrasG12D cooperate to promote chromosomal instability and widely metastatic pancreatic ductal adenocarcinoma in mice. Cancer Cell *7*, 469–483.

Hong, A., Moriceau, G., Sun, L., Lomeli, S., Piva, M., Damoiseaux, R., Holmen, S.L., Sharpless, N.E., Hugo, W., and Lo, R.S. (2018). Exploiting drug addiction mechanisms to select against MAPKi-resistant melanoma. Cancer Discov. *8*, 74–93.

Hruban, R.H., Adsay, N.V., Albores-Saavedra, J., Compton, C., Garrett, E.S., Goodman, S.N., Kern, S.E., Klimstra, D.S., Kloppel, G., Longnecker, D.S., et al. (2001). Pancreatic intraepithelial neoplasia: a new nomenclature and classification system for pancreatic duct lesions. Am. J. Surg. Pathol. *25*, 579–586.

Hruban, R.H., Adsay, N.V., Albores-Saavedra, J., Anver, M.R., Biankin, A.V., Boivin, G.P., Furth, E.E., Furukawa, T., Klein, A., Klimstra, D.S., et al. (2006). Pathology of genetically engineered mouse models of pancreatic exocrine cancer: consensus report and recommendations. Cancer Res. *66*, 95–106.

Ishikawa, K., Takenaga, K., Akimoto, M., Koshikawa, N., Yamaguchi, A., Imanishi, H., Nakada, K., Honma, Y., and Hayashi, J. (2008). ROS-generating mitochondrial DNA mutations can regulate tumor cell metastasis. Science *320*, 661–664.

Jiang, L., Shestov, A.A., Swain, P., Yang, C., Parker, S.J., Wang, Q.A., Terada, L.S., Adams, N.D., McCabe, M.T., Pietrak, B., et al. (2016). Reductive carboxylation supports redox homeostasis during anchorage-independent growth. Nature *532*, 255–258.

Joneson, T., and Bar-Sagi, D. (1999). Suppression of Ras-induced apoptosis by the rac GTPase. Mol. Cell Biol. *19*, 5892–5901.

Jonkers, J., Meuwissen, R., van der Gulden, H., Peterse, H., van der Valk, M., and Berns, A. (2001). Synergistic tumor suppressor activity of BRCA2 and p53 in a conditional mouse model for breast cancer. Nat. Genet. *29*, 418–425.

Klein, E.A., Thompson, I.M., Jr., Tangen, C.M., Crowley, J.J., Lucia, M.S., Goodman, P.J., Minasian, L.M., Ford, L.G., Parnes, H.L., Gaziano, J.M., et al. (2011). Vitamin E and the risk of prostate cancer: the selenium and vitamin E cancer prevention trial (SELECT). JAMA *306*, 1549–1556.

Krebs, A.M., Mitschke, J., Lasierra Losada, M., Schmalhofer, O., Boerries, M., Busch, H., Boettcher, M., Mougiakakos, D., Reichardt, W., Bronsert, P., et al. (2017). The EMT-activator Zeb1 is a key factor for cell plasticity and promotes metastasis in pancreatic cancer. Nat. Cell Biol. *19*, 518–529.

Labuschagne, C.F., Cheung, E.C., Blagih, J., Domart, M.C., and Vousden, K.H. (2019). Cell clustering promotes a metabolic switch that supports metastatic colonization. Cell Metab. *30*, 720–734.e5.

Le Gal, K., Ibrahim, M.X., Wiel, C., Sayin, V.I., Akula, M.K., Karlsson, C., Dalin, M.G., Akyurek, L.M., Lindahl, P., Nilsson, J., et al. (2015). Antioxidants can increase melanoma metastasis in mice. Sci. Transl. Med. *7*, 308re308.

Lee, P., Vousden, K.H., and Cheung, E.C. (2014). TIGAR, TIGAR, burning bright. Cancer Metab. *2*, 1.

Li, M., Sun, M., Cao, L., Gu, J.H., Ge, J., Chen, J., Han, R., Qin, Y.Y., Zhou, Z.P., Ding, Y., et al. (2014). A TIGAR-regulated metabolic pathway is critical for protection of brain ischemia. J. Neurosci. *34*, 7458–7471.

Lignitto, L., LeBoeuf, S.E., Homer, H., Jiang, S., Askenazi, M., Karakousi, T.R., Pass, H.I., Bhutkar, A.J., Tsirigos, A., Ueberheide, B., et al. (2019). Nrf2 activation promotes lung cancer metastasis by inhibiting the degradation of Bach1. Cell *178*, 316–329.e18.

Liou, G.Y., Doppler, H., DelGiorno, K.E., Zhang, L., Leitges, M., Crawford, H.C., Murphy, M.P., and Storz, P. (2016). Mutant KRas-induced mitochondrial oxidative stress in acinar cells upregulates EGFR signaling to drive formation of pancreatic precancerous lesions. Cell Rep. *14*, 2325–2336.

Lu, W., and Kang, Y. (2019). Epithelial-mesenchymal plasticity in cancer progression and metastasis. Dev. Cell *49*, 361–374.

Maddocks, O.D.K., Athineos, D., Cheung, E.C., Lee, P., Zhang, T., van den Broek, N.J.F., Mackay, G.M., Labuschagne, C.F., Gay, D., Kruiswijk, F., et al. (2017). Modulating the therapeutic response of tumours to dietary serine and glycine starvation. Nature *544*, 372–376.

Mitsuishi, Y., Taguchi, K., Kawatani, Y., Shibata, T., Nukiwa, T., Aburatani, H., Yamamoto, M., and Motohashi, H. (2012). Nrf2 redirects glucose and glutamine into anabolic pathways in metabolic reprogramming. Cancer Cell *22*, 66–79.

Muda, M., Boschert, U., Dickinson, R., Martinou, J.C., Martinou, I., Camps, M., Schlegel, W., and Arkinstall, S. (1996). MKP-3, a novel cytosolic protein-tyrosine phosphatase that exemplifies a new class of mitogen-activated protein kinase phosphatase. J. Biol. Chem. *271*, 4319–4326.

O'Leary, B.R., Fath, M.A., Bellizzi, A.M., Hrabe, J.E., Button, A.M., Allen, B.G., Case, A.J., Altekruse, S., Wagner, B.A., Buettner, G.R., et al. (2015). Loss of SOD3 (EcSOD) expression promotes an aggressive phenotype in human pancreatic ductal adenocarcinoma. Clin. Cancer Res. *21*, 1741–1751.

Pang, L., Sawada, T., Decker, S.J., and Saltiel, A.R. (1995). Inhibition of MAP kinase kinase blocks the differentiation of PC-12 cells induced by nerve growth factor. J. Biol. Chem. *270*, 13585–13588.

Piskounova, E., Agathocleous, M., Murphy, M.M., Hu, Z., Huddlestun, S.E., Zhao, Z., Leitch, A.M., Johnson, T.M., DeBerardinis, R.J., and Morrison, S.J. (2015). Oxidative stress inhibits distant metastasis by human melanoma cells. Nature *527*, 186–191.

Porporato, P.E., Payen, V.L., Perez-Escuredo, J., De Saedeleer, C.J., Danhier, P., Copetti, T., Dhup, S., Tardy, M., Vazeille, T., Bouzin, C., et al. (2014). A mitochondrial switch promotes tumor metastasis. Cell Rep. *8*, 754–766.

Radisky, D.C., Levy, D.D., Littlepage, L.E., Liu, H., Nelson, C.M., Fata, J.E., Leake, D., Godden, E.L., Albertson, D.G., Nieto, M.A., et al. (2005). Rac1b and reactive oxygen species mediate MMP-3-induced EMT and genomic instability. Nature *436*, 123–127.

Rojo de la Vega, M., Chapman, E., and Zhang, D.D. (2018). NRF2 and the hallmarks of cancer. Cancer Cell *34*, 21–43.

Saikolappan, S., Kumar, B., Shishodia, G., Koul, S., and Koul, H.K. (2019). Reactive oxygen species and cancer: a complex interaction. Cancer Lett. *452*, 132–143.

Sayin, V.I., Ibrahim, M.X., Larsson, E., Nilsson, J.A., Lindahl, P., and Bergo, M.O. (2014). Antioxidants accelerate lung cancer progression in mice. Sci. Transl. Med. *6*, 221ra215.

Schafer, Z.T., Grassian, A.R., Song, L., Jiang, Z., Gerhart-Hines, Z., Irie, H.Y., Gao, S., Puigserver, P., and Brugge, J.S. (2009). Antioxidant and oncogene rescue of metabolic defects caused by loss of matrix attachment. Nature *461*, 109–113.

Schieber, M., and Chandel, N.S. (2014). ROS function in redox signaling and oxidative stress. Curr. Biol. *24*, R453–R462.

Schriner, S.E., Linford, N.J., Martin, G.M., Treuting, P., Ogburn, C.E., Emond, M., Coskun, P.E., Ladiges, W., Wolf, N., Van Remmen, H., et al. (2005).

Extension of murine life span by overexpression of catalase targeted to mitochondria. Science *308*, 1909–1911.

Shen, M., Zhao, X., Zhao, L., Shi, L., An, S., Huang, G., and Liu, J. (2018). Met is involved in TIGAR-regulated metastasis of non-small-cell lung cancer. Mol. Cancer *17*, 88.

Tafani, M., Sansone, L., Limana, F., Arcangeli, T., De Santis, E., Polese, M., Fini, M., and Russo, M.A. (2016). The interplay of reactive oxygen species, hypoxia, inflammation, and sirtuins in cancer initiation and progression. Oxid. Med. Cell Longev. *2016*, 3907147.

ten Kate, M., van der Wal, J.B., Sluiter, W., Hofland, L.J., Jeekel, J., Sonneveld, P., and van Eijck, C.H. (2006). The role of superoxide anions in the development of distant tumour recurrence. Br. J. Cancer *95*, 1497–1503.

Tsai, J.H., Donaher, J.L., Murphy, D.A., Chau, S., and Yang, J. (2012). Spatiotemporal regulation of epithelial-mesenchymal transition is essential for squamous cell carcinoma metastasis. Cancer Cell *22*, 725–736.

Weinberg, F., Hamanaka, R., Wheaton, W.W., Weinberg, S., Joseph, J., Lopez, M., Kalyanaraman, B., Mutlu, G.M., Budinger, G.R., and Chandel, N.S. (2010). Mitochondrial metabolism and ROS generation are essential for Kras-mediated tumorigenicity. Proc. Natl. Acad. Sci. U S A *107*, 8788–8793.

Wiel, C., Le Gal, K., Ibrahim, M.X., Jahangir, C.A., Kashif, M., Yao, H., Ziegler, D.V., Xu, X., Ghosh, T., Mondal, T., et al. (2019). BACH1 stabilization by antioxidants stimulates lung cancer metastasis. Cell *178*, 330–345.e22.

Wipf, P., Xiao, J., Jiang, J., Belikova, N.A., Tyurin, V.A., Fink, M.P., and Kagan, V.E. (2005). Mitochondrial targeting of selective electron scavengers: synthesis and biological analysis of hemigramicidin-TEMPO conjugates. J. Am. Chem. Soc. *127*, 12460–12461.

Woo, D.K., Green, P.D., Santos, J.H., D'Souza, A.D., Walther, Z., Martin, W.D., Christian, B.E., Chandel, N.S., and Shadel, G.S. (2012). Mitochondrial genome instability and ROS enhance intestinal tumorigenesis in APC(Min/+) mice. Am. J. Pathol. *180*, 24–31.

Woods, D., Parry, D., Cherwinski, H., Bosch, E., Lees, E., and McMahon, M. (1997). Raf-induced proliferation or cell cycle arrest is determined by the level of Raf activity with arrest mediated by p21Cip1. Mol. Cell Biol. *17*, 5598–5611.

Yang, W., Zou, L., Huang, C., and Lei, Y. (2014). Redox regulation of cancer metastasis: molecular signaling and therapeutic opportunities. Drug Dev. Res. *75*, 331–341.

Ying, H., Kimmelman, A.C., Lyssiotis, C.A., Hua, S., Chu, G.C., Fletcher-Sananikone, E., Locasale, J.W., Son, J., Zhang, H., Coloff, J.L., et al. (2012). Oncogenic Kras maintains pancreatic tumors through regulation of anabolic glucose metabolism. Cell *149*, 656–670.

Zheng, X., Carstens, J.L., Kim, J., Scheible, M., Kaye, J., Sugimoto, H., Wu, C.C., LeBleu, V.S., and Kalluri, R. (2015). Epithelial-to-mesenchymal transition is dispensable for metastasis but induces chemoresistance in pancreatic cancer. Nature *527*, 525–530.

STAR★METHODS

KEY RESOURCES TABLE

REAGENT or RESOURCE	SOURCE	IDENTIFIER
Antibodies		
Rabbit polyclonal anti-TIGAR	Santa Cruz	cat # sc67273; RRID: AB_1128224
Rabbit monoclonal anti-p-ERK1/2 Thr202/204	Cell Signaling	cat # 9101S; RRID: AB_331646
Rabbit monoclonal anti-DUSP6	Abcam	Cat # Ab76310; RRID: AB_1523517
Rabbit monoclonal anti-Snail	Cell Signaling	Cat # 3879; RRID: AB_2255011
Rabbit monoclonal anti-Slug	Cell Signaling	Cat # 9585; RRID: AB_2239535
Rabbit monoclonal anti-E-Cadherin	Cell Signaling	Cat # 3195; RRID: AB_2291471
Rabbit polyclonal anti-p-FAK Tyr576/577	Cell Signaling	Cat # 3281; RRID: AB_331079
Rabbit monoclonal anti-p-Akt Ser 473	Cell Signaling	Cat # 13038; RRID: AB_2629447
Rabbit monoclonal anti-GCLC	Abcam	Cat # Ab190685
Rabbit polyclonal anti-HO1	Santa Cruz	Cat # sc10789; RRID: AB_648281
Rabbit monoclonal anti p-NF-kB p65	Cell Signaling	Cat # 3033; RRID: AB_331284
Rabbit monoclonal anti-p-Src family Tyr416	Cell Signaling	Cat # 6943; RRID: AB_10013641
Rabbit polyclonal anti-p-Stat3 Tyr 705	Cell Signaling	Cat # 9145; RRID: AB_2491009
Mouse monoclonal anti-Actin HRP conjugated	Abcam	Cat # Ab20272; RRID: AB_445482
Rabbit polyclonal anti-ERK	Cell Signaling	Cat # 9102; RRID: AB_330744
Rabbit polyclonal anti-BNIP3	Cell Signaling	Cat # 3769; RRID: AB_2259284
Rabbit monoclonal anti-MET	Cell Signaling	Cat # 8198; RRID: AB_10858224
Mouse monoclonal anti-BACH1	Santa Cruz	Cat # sc271211; RRID: AB_10608972
Mouse monoclonal anti-TIGAR	Bensaad et al. (2006)	NA
Rabbit monoclonal anti-Ki67	Thermo Scientific	Cat # SP6; RRID: AB_10979488
Rabbit polyclonal anti-MDA	Abcam	Cat # Ab6463; RRID: AB_305484
Rabbit polyclonal anti-TIGAR	Millipore	Cat # AB10545; RRID: AB_10807181
Rabbit monoclonal anti-Vimentin	Cell Signaling	Cat # 5741; RRID: AB_10695459
Rabbit monoclonal Anti-Cytokeratin 19 (CK-19)	Abcam	Cat # Ab52625; RRID: AB_2281020
Rabbit polyclonal anti-Histone H3	Cell Signaling	Cat # 9715; RRID: AB_331563
Mouse monoclonal Anti-E-Cadherin	BD Biosciences	Cat # 610182; RRID: AB_397581
Biological Samples		
Tissue Microarray of human pancreatic cancer	US Biomax	Cat # PA2081b
Chemicals, Peptides, and Recombinant Proteins		
Adriamycin	Sigma	cat # D1515
N-acetyl-L-Cysteine (NAC)	Sigma	cat # A7250
Recombinant TIGAR-TAT (rTIGAR)	Peprotech	cat # 150-14T
Antimycin A	Caymen Chemical	cat # 19433
PD98059	Tocris	cat # 1213
MitoSOX™ Red	Thermo Fisher Scientific	cat #M36008
Mito-TEMPO	Sigma	cat # SML0737
Piericidin	Caymen Chemical	cat # 15379
Critical Commercial Assays		
Invadopodia assay (QCM™ Gelatin Invadopodia Assay)	Millipore	cat # ECM 670
Migration assay (BioCoat™ Control Insert-No ECM, 8 micron pore size)	Corning®	Cat # 354578
Invasion assays (BioCoat™ Matrigel Invasion chamber)	Corning®	Cat # 354480
LIVE/DEAD Viability/Cytotoxicity Kit	Thermo Fisher Scientific	Cat # L3224

(*Continued on next page*)

Continued		
REAGENT or RESOURCE	SOURCE	IDENTIFIER
Experimental Models: Cell Lines		
Mouse PDAC cell lines	This paper	NA
Experimental Models: Organisms/Strains		
Mouse: athymic *nu/nu* mice	The Jackson Laboratory	002019
Mouse: *Pdx1-Cre; Kras*$^{+/LSL\text{-}G12D}$;*Trp53*$^{+/LSL\text{-}R172H}$	Hingorani et al. (2005)	NA
Mouse: *TIGAR*$^{fl/fl}$	Cheung et al. (2016)	NA
Mouse: *Pdx1-Cre; Kras*$^{+/LSL\text{-}G12D}$; *Trp53*$^{+/LSL\text{-}R270H}$; *Nrf2*$^{-/-}$	DeNicola et al. (2011)	NA
Mouse: *Trp53*$^{fl/fl}$	Jonkers et al. (2001)	NA
Oligonucleotides		
siRNA targeting ERK1/2 and corresponding non targeting controls	Origene	cat# SR412074, cat# 412814
Recombinant DNA		
pCMV6-Entry DUSP6 Myc-DDK tagged	Origene	cat# MR222688
pCMV6-Entry Myc-DDK tagged empty vector	This paper	NA
Software and Algorithms		
Prism 7	GraphPad	https://www.graphpad.com/scientific-software/prism/; RRID: SCR_002798
TraceFinder Version 4.1	Thermo Fisher Scientific	OPTON-30626
ImageJ	https://doi.org/10.1038/nmeth.2089	https://imagej.nih.gov/ij/

LEAD CONTACT AND MATERIALS AVAILABILITY

The Lead Contact is Karen H Vousden (karen.vousden@crick.ac.uk). All unique/stable reagents generated in this study are available from the Lead Contact with a completed MTA. Further information and requests for resources and reagents should be directed to and will be fulfilled by the Lead Contact Karen H Vousden (karen.vousden@crick.ac.uk).

EXPERIMENTAL MODEL AND SUBJECT DETAILS

In Vivo Animal Studies

All animal experiments were performed under the Animals (Scientific Procedures) Act 1986 in accordance with UK Home Office licenses (Project License 70/8645, P319AE968) and the EU Directive 2010 and sanctioned by local ethical review process (University of Glasgow and The Francis Crick Institute). Mice were housed in an area free of pathogens as defined by FELASA recommendations in IVC ages at 5 per cage at constant temperature (19-23°C) and humidity (55% ± 10%), with a 12-hour light/dark cycle (lights on at 7:00 am) and were allowed access to food and water *ad libitum*. Mice were allowed to acclimatize for at least 2 days (for mice bred on site) or 7 days (for imported mice) prior to the experiment and were randomly assigned to experimental groups. For the PDAC GEMMs, both male and female mice were used, roughly matched between CTR and KO groups. For the tail vein lung colonization models only female mice were used. Mice had not been involved in any previous procedures.

Transgenic Mouse Models for Pancreatic Ductal Adenocarcinoma (PDAC)

Trp53$^{+/LSL\text{-}R172H}$, *Kras*$^{+/LSL\text{-}G12D}$, *Trp53*$^{+/fl}$, *Pdx1-Cre* strains were interbred to obtain KC (*Pdx1-Cre; Kras*$^{+/LSL\text{-}G12D}$), KFC (*Pdx1-Cre; Kras*$^{+/LSL\text{-}G12D}$; *Trp53*$^{+/fl}$), and KPC (*Pdx1-Cre; Kras*$^{+/LSL\text{-}G12D}$; *Trp53*$^{+/LSL\text{-}R172H}$) mice (Hingorani et al., 2005; Jonkers et al., 2001). To introduce TIGAR deficiency in these models, *TIGAR*$^{fl/fl}$ strain (Cheung et al., 2016) was used to breed into the above strains to obtain *Tigar*$^{fl/+}$ or *Tigar*$^{+/+}$ for control (CTR) and *Tigar*$^{fl/fl}$ for *Tigar* knockout (KO) in KC, KFC and KPC in a mixed background. *Pdx1-Cre; Kras*$^{+/LSL\text{-}G12D}$; *Trp53*$^{+/LSL\text{-}R270H}$; *Nrf2*$^{-/-}$ (DeNicola et al., 2011) mice were also used. Mice were monitored two times weekly and tissues were collected when exhibiting symptoms of PDAC (Hingorani et al., 2005).

Lung Metastasis Model

2X10^5 PDAC KFC cells per mouse (n$\geq$4 in each group) in 100μl PBS were injected (tail vein) into athymic *nu/nu* mice (Jackson Laboratory). After 14 days, lung tissues were collected for histological analysis. For antioxidant treatment, a week before the injection NAC (N-Acetyl-L-cysteine, Sigma A7250) was administered to the mouse (1g/L drinking water, pH 7) and throughout the duration of experiment. PDAC cells were pre-treated overnight with NAC before trypsinized and used at the day of injection.

Cell Cultures

PDAC tumor cell lines were derived from the KFC tumors from three TIGAR WT and three TIGAR KO animals. Tumor tissues were collected in PBS with 1% penicillin-streptomycin and then finely minced. Minced tissues were then incubated with collagenase type 1 (200U/ml, Gibco) and dispase (2.4U/ml, Gibco) in HBSS for 1 hour in 37°C for cell dissociation. After washing 2X in HBSS, cell pellets were resuspended and grown in growth media (Dulbecco's modified Eagle medium containing 10% fetal bovine serum, 2 mM l-glutamine, 1% penicillin-streptomycin).

METHOD DETAILS

Transwell Migration/Invasion Assays

Transwell Migration assays (Corning® BioCoat™ Control Insert-No ECM, 8 micron pore size) and Invasion assays (Corning® BioCoat™ Matrigel Invasion chamber) were performed according to the manufacturer's instructions. Briefly, cells were pre-treated overnight in 1% serum with or without the indicated drugs/treatments before seeding onto the upper chamber of the transwell the following day in the presence of the drug treatment. Media with 10% serum (with and without the drugs) were used in the lower chamber. After 16 hours, cells that remained on the top of the membrane were removed by a cotton-tipped applicator. Cell that were migrated/invaded to the bottom of the insert were then fixed in 70% ethanol and stained with 0.5% Crystal Violet. Migrated/Invaded cells were photographed under an inverted microscope, quantified using ImageJ, and represented as percentage of total area.

Wound Scratch Assay

Confluent monolayer of cells was scratched using a p20 pipette tip to create a scratch. Debris were removed by washing the cells gently with 2X complete media. Image were taken at the start of the assay and after 16 hours under a phase contrast microscope. The width of the gap was measured by ImageJ and the reduction of the width is represented as percentage (%) wound closure.

Histology and Immunohistochemistry

Tissues were fixed in 10% neutral buffered formalin and were embedded in paraffin and processed by standard histological techniques. PanIN was defined by previously published guildelines in genetically modified mouse models of PDAC. Briefly, PanIN is a lesion that arises in native pancreatic ducts measuring <1 mm and not on a background of acinar-ductal metaplasia. PanIN lesions are graded as PanIN-1, 2 or 3 according to the cytological and architectural characteristics as described previously (Hruban et al., 2001). Quantitation of PanIN number was done on 5 of 20X fields of view from at least 4 mice. Heat induced epitope retrieval with sodium citrate buffer (Antigen Unmasking Solution, Citric Acid Based, Vector Laboratories, cat # H-3300) followed by blocking endogenous peroxidase and Avidin/Biotin (BLOXALL Endogenous Peroxidase and Alkaline Phosphatase Blocking Solution, Vector Laboratories, cat # SP-6000) were used for immunohistochemistry prior to primary antibody incubation (diluted in 10% normal horse serum in 1XTBST, 4°C overnight). For immunohistochemistry, primary antibodies used were anti-Ki67 (1:1000 Thermo Scientific SP6), anti-MDA (1:300, Abcam Ab6463), anti-TIGAR (1:500 Millipore AB10545), anti-phospho-ERK (Cell Signalling), anti-DUSP6 (1:300 Abcam Ab76310), anti-Snail (1:300 Cell Signalling #3879), anti-Slug (1:300 Cell Signalling #9585), anti-E-Cadherin (1:300 Cell Signalling), anti-Vimentin (1:300, Cell Signaling #5741), anti-Cytokeratin 19 (CK-19) (1:500, Abcam Ab52625). Expression levels were scored based on staining intensity and area of tumor cells using a weighted histoscore calculated from the sum of (1 × % weak staining) + (2 × % moderate staining) + (3 × % strong staining).

Human TMA Analysis

TMA of human pancreatic cancer was obtained from US Biomax (PA2081b) and was stained with anti-human TIGAR antibody (Bensaad et al., 2006).

Cell Death, ROS Measurement, and Western Blot Analysis

Cell death was quantified using LIVE/DEAD Viability Kit (Molecular Probes) 18 hours after adriamycin (1μg/ml, Sigma) alone or with either NAC (1mM) or recombinant TIGAR (rTIGAR, 5μg/ml, Peprotech). Mito tempo (50μM, Sigma SML0737), Piericidin (1μM, Caymen Chemical 15379), Antimycin A (1μM, Caymen Chemical 19433) and PD98059 (50μM, Tocris cat# 1213) were used at the indicated times and duration. Mitochondrial ROS was measured by MitoSOX™ Red Mitochondrial Superoxide Indicator (Invitrogen cat # M36008). Protein lysates were isolated in RIPA-buffer (Millipore) with complete protease inhibitors (Roche) and phosphatase inhibitor cocktail (Thermo Fisher Scientific), volume adjusted according to protein concentration measurements (Quick Start™ Bradford 1X Dye Reagent, Bio-Rad, Cat #500-0205), separated using precast NuPAGE 4-12% Bis-Tris Protein gels (Invitrogen, Life Technologies), and transferred to nitrocellulose membranes. ECL chemiluminescence detection kits (Pierce) with appropriate species-specific horseradish peroxidase−conjugated secondary antibodies were used to detect the proteins. Each blot shows one representative out of at least three. Antibodies used are : anti-TIGAR (1:1000, Santa Cruz sc-67273), anti-p-ERK1/2 Thr202/204 (1:1000, Cell Signaling), anti-DUSP6 (1: 500 Abcam Ab76310), anti-Snail (1:1000, Cell Signaling #3879), anti-Slug (1:1000, Cell Signaling #9585), anti-E-Cadherin (1:5000, Cell Signaling #3195), anti-p-FAK Tyr576/577 (1:1000, Cell Signaling #3281), anti-p-Akt Ser 473 (1:1000, Cell Signaling #13038), anti-GCLC (1:1000 Abcam Ab190685), anti-HO1 (1:500, Santa Cruz sc10789), anti p-NF-kB p65 (1:1000, Cell Signaling #3033), anti-p-Src family (1:500, Cell Signaling #6943), anti-p-Stat3 Tyr 705 (1:1000, Cell Signaling #9145), anti-Actin (1:10000, Abcam Ab20272), anti-ERK (1:1000, Cell Signaling #9102), anti-BACH1 (1:1000, Santa Cruz sc-271211), anti-BNIP3 (1:1000, Cell

Signaling #3769), anti-MET (1:1000, Cell Signaling #8198), anti-Vinculin (1:1000, Santa Cruz sc-25336), anti-Histone H3 (1:1000, Cell Signaling #9715).

Transfection of siRNA and cDNA

Re-expression of DUSP6 was achieved by transfecting a mouse tagged ORF clone of DUSP6 (Origene, cat#MR222688) with empty vector as control. Knockdown of ERK1/2 was achieved by transfecting siRNA targeting ERK1/2 (Origene, cat#SR412074, 412814) with the corresponding scrambled siRNA as negative control. Transfection was performed using Lipofectamine 2000 for DNA (Invitrogen) or Lipofectamine RNAiMAX for siRNA (Invitrogen) according to the manufacturer's instructions.

Immunofluorescence Staining

Cells were fixed in 4% paraformaldehyde in 1X PBS, followed by permeabilization with 0.4% Triton X 100 in 1XPBS with 10% normal donkey serum. Primary antibody was prepared in 1XPBS with 0.4% Triton X-100 and 2% normal donkey serum. Antibody used were E-Cadherin (1:500, BD Biosciences, #610182) and Vimentin (1:500, Cell Signaling, #5741). Fixed cells were incubated overnight in 4°C with the primary antibody, washed 3X in 1XPBS, followed by incubation of secondary antibody for 1 hour at room temperature (1:500 in 1XPBS with 0.4% Triton X-100 and 2% normal donkey serum, Alexa Fluor 488 donkey anti-mouse Alexa Fluor 488 or Alexa Fluor 594 donkey anti-rabbit, Thermo Fischer Scientific).

Invadopodia Assay

Invadopodia assay (QCM^{TM} Gelatin Invadopodia Assay, Millipore) was performed according to the manufacturer's instructions. Briefly, glass chamber slides were coated with Poly L Lysine, activated by a diluted glutaldehyde solution, and then fluorescently coated with fluorescent gelatin as the substrate for invadopodia. After disinfecting with 70% alcohol and quenching of free aldehydes with growth medium, cells were seeded onto the gelatin surface for 16 hours in complete media. Cells were then fixed in 4% formaldehyde in DPBS and visualized by nuclear (DAPI) and cytoskeleton staining (TRITC-Phalloidin) by fluorescent microscopy. Degraded area/puncta of fluorescent gelatin (devoid of green fluorescence) indicated the presence of invadopodia and was quantified per number of cells (at least 100 cells were analysed per experiment).

Metabolomics

Cells were seeded in 6 well plates at a density of $1x10^5$ in DMEM. The media was refreshed after 24 hours and replaced after 48 hours with media containing 1,2-^{13}C2-glucose. Cells were cultured in this media for 5 hours before harvesting the cells for metabolomics and isotope tracing analysis. Cells were harvested by removing the media and rinsing with cold PBS before cells were lysed by adding cold (-20°C) extraction buffer containing methanol, acetonitrile and water (50:30:20) directly on the cells followed by scraping and collecting everything in a clean 1.5 ml centrifuge tube. The tubes were vortexed for 1 min before centrifugation at full speed for 15 minutes at 4°C. The supernatant was collected into mass spectrometry tubes and analysed by liquid chromatography high resolution mass spectrometry as described before (Labuschagne et al., 2019). Briefly, liquid chromatography was performed on a Dionex Ultimate 3000 LC system coupled to a Q Exactive mass spectrometer (Thermo Scientific). Metabolites were separated on a Sequant ZIC-pHILIC column (2.1 x 150 mm, 5mM) (Merck) with mobile phase A consisting of 20 mM (NH4)2CO3, 0.1% NH4OH in H2O and mobile phase B consisting of 100% acetonitrile. A linear gradient from 80% to 20% A was applied over 17 minutes at a flow rate of 200 ul/min. Eluents were ionized in a HESI probe connected to the Q Exactive which scanned a mass range between 75 and 1000 m/z with polarity switching. Data were analysed with using Thermo TraceFinder software.

QUANTIFICATION AND STATISTICAL ANALYSIS

Data were analysed using GraphPad Prism 7 software (GraphPad Software). The survival data were analysed by log-rank Mantel-Cox test. Fisher's exact test was used to compare frequency of metastasis. Other data represent mean values ± SEM from at least three independent experiments ($n \geq 3$). Student's *t* test (comparisons between two groups), one-way ANOVA with Tukey post hoc (comparisons of three or more groups with one independent variable) were used as indicated in the legends. $p < 0.05$ was considered statistically significant.

DATA AND CODE AVAILABILITY

This study did not generate any unique datasets or code.

Cancer Cell, Volume *37*

Supplemental Information

Dynamic ROS Control by TIGAR Regulates the Initiation and Progression of Pancreatic Cancer

Eric C. Cheung, Gina M. DeNicola, Colin Nixon, Karen Blyth, Christiaan F. Labuschagne, David A. Tuveson, and Karen H. Vousden

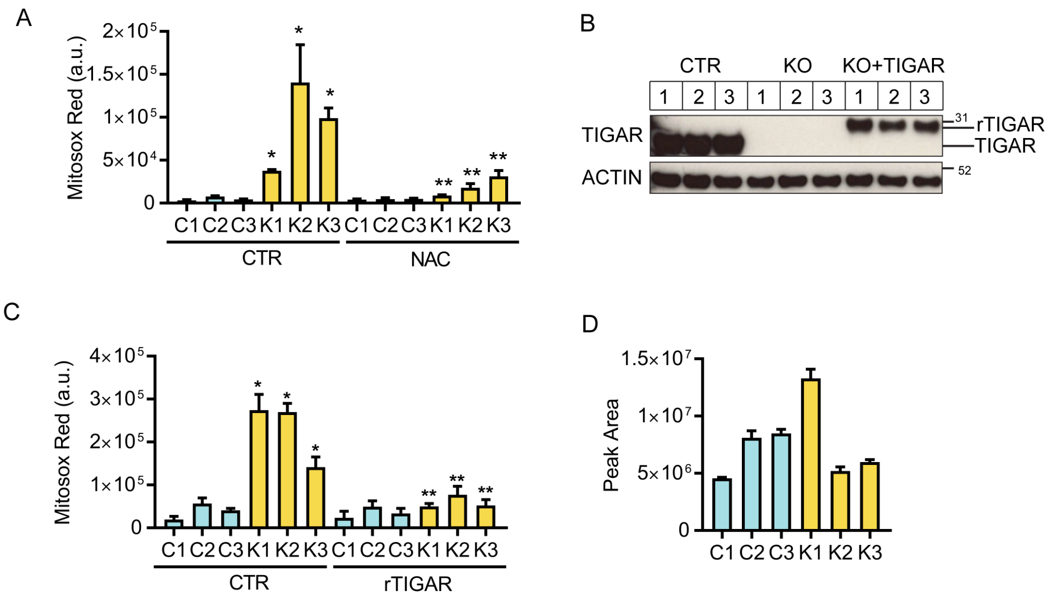

Figure S1. Related to Figure 1. Regulation of mitochondrial ROS by TIGAR (A) Mitochondrial ROS measurement of CTR (C1-3) and TIGAR KO (K1-3) KFC PDAC cells with or without (CTR, no treatment) NAC. * *p<0.05* K1-3 compared to C1-C3, ** *p<0.05* NAC treated K1-3 compared to CTR treated K1-3. (B) Representative Western blot analysis of one set of samples from (C) (CTR, KO and KO+TIGAR KFC PDAC cells) treated with recombinant TIGAR (rTIGAR). ACTIN was used as loading control. (C) Mitochondrial ROS measurement of CTR and KO KFC PDAC cells with or without (CTR, no treatment) rTIGAR. * *p<0.05* K1-3 compared to C1-3, ** *p<0.05* K1-3 with rTIGAR compared to CTR treated K1-3. (D) Mass spectroscopy measurement of ribose 5 phosphate of CTR and KO KFC PDAC cells. (A,C,D) Error bars represent mean ± SEM, and data analysed by one way ANOVA with Tukey post hoc test. n=3 independent experiments for each cell line.

A

KPC

Mouse ID	Sex	Cohort	Histology 1°	Histology 2°
163208	M	HET	U	
173269	F	HET	G	MD
173271	F	HET	G	C
174844	F	HET	G	
177969	M	HET	G	U
182986	F	HET	G	
183004	M	HET	G	MD
183006	M	WT	U	
183010	F	HET	G	
183014	F	HET	G	
185096	M	HET	G	U
185105	F	HET	G	
186253	M	HET	G	U
186757	F	HET	U	
188464	F	HET	G	U
190411	F	HET	G	
190413	F	HET	G	U
190414	F	HET	G	U
190421	M	WT	G	U
194350	M	HET	G	U
197103	F	WT	G	U
197113	F	HET	G	
197115	F	HET	U	
197412	F	WT	U	
197414	F	WT	G	
199661	M	HET	G	U
199662	M	HET	G	U
199664	M	HET	G	U
200861	F	WT	G	U
203183	M	WT	G	U
200858	M	WT	G	C
185109	F	WT	MD	U
190422	F	WT	MD	U

Mouse ID	Sex	Cohort	Histology 1°	Histology 2°
163230	F	KO	G	MD
173265	M	KO	G	MD
173921	M	KO	G	U
174843	F	KO	G	MD
178357	F	KO	G	U
178631	F	KO	U	
182052	M	KO	G	MD
182059	F	KO	G	
182990	M	KO	G	
182991	M	KO	G	U
182997	F	KO	G	MD
183869	F	KO	G	MD
183870	F	KO	G	C
186259	F	KO	G	
186746	M	KO	G	
186748	M	KO	G	C
187160	F	KO	G	
187162	M	KO	G	
187163	M	KO	G	
187167	M	KO	G	C
187168	F	KO	G	U
187169	F	KO	G	C
187170	F	KO	G	U
188467	F	KO	G	
190404	M	KO	G	
190407	F	KO	G	U
190972	F	KO	G	
193646	F	KO	G	
193662	M	KO	G	
193663	M	KO	G	
196868	F	KO	G	
198041	M	KO	G	
202762	M	KO	G	U

B

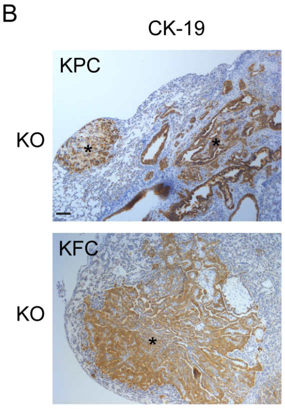

C

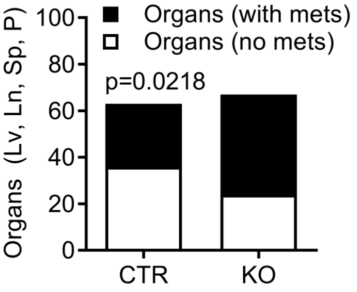

KFC

Mouse ID	Sex	Cohort	Histology 1°	Histology 2°
OECF15.1f	F	HET	S	U
OECF13.1a	F	HET	U	S
OECF13.1g	F	HET	Norm	G
OECF14.1f	M	HET	Norm	G
OECF20.1c	M	HET	MD	S
OECF15.3c	F	HET	MD	Norm
OECF19.2a	F	HET	Norm	G
OECF19.1d	F	HET	S	G
OECF21.1b	F	HET	G	MD
OECF17.2c	F	HET	U	S
OECF14.4d	M	HET	S	C
OECF15.3a	F	HET	G	MD
OECF20.2h	F	HET	G	MD
OECF22.1c	F	HET	MD	Norm
OECF20.3d	F	HET	G	MD
OECF14.6c	F	WT	Norm	G
OECF28.4f	M	HET	C	U
OECF22.2c	M	HET	G	MD
OECF20.4d	M	HET	U	MD
OECF20.4f	M	HET	C	C
OECF31.1a	F	WT	Norm	MD
OECF32.1c	M	WT	U	
OECF32.1f	M	WT	U	
OECF32.1g	M	WT	U	G
OECF19.5c	M	HET	U	S
OECF19.5f	M	WT	U	S
OECF32.2b	M	WT	MD	S
OECF29.5g	F	WT	G	S
OECF29.6c	M	WT	G	MD
OECF36.1d	M	WT	S	G
OECF36.2b	M	WT	U	S
OECF36.3f	F	WT	MD	S

Mouse ID	Sex	Cohort	Histology 1°	Histology 2°
OECF16.1b	M	KO	S	U
OECF16.1f	M	KO	G	S
OECF16.1g	M	KO	U	S
OECF15.1c	F	KO	S	G
OECF13.1d	F	KO	Norm	C
OECF16.2a	M	KO	Norm	G
OECF19.1a	M	KO	G	U
OECF17.1g	F	KO	C	C
OECF15.3f	F	KO	MD	G
OECF15.3g	F	KO	U	S
OECF20.2g	F	KO	MD	S
OECF27.2c	M	KO	MD	G
OECF27.2d	M	KO	MD	S
OECF28.3d	M	KO	Norm	MD
OECF28.3i	F	KO	G	MD
OECF13.2g	M	KO	U	
OECF26.3c	F	KO	U	Norm
OECF20.2e	F	KO	Norm	G
OECF14.1g	M	KO	Norm	G
OECF15.2a	M	KO	Norm	G
OECF19.4g	F	KO	U	Norm
OECF17.4e	F	KO	U	S
OECF17.4f	F	KO	U	MD
OECF27.3e	M	KO	G	Norm
OECF28.5a	M	KO	Norm	MD
OECF19.5g	F	KO	S	C
OECF19.5j	F	KO	U	MD

D

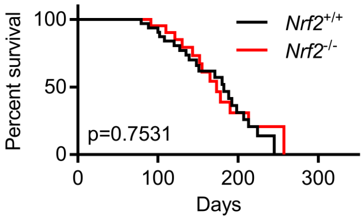

Figure S2. Related to Figure 2. *Tigar* deletion in KRAS driven pancreatic ductal adenocarcinoma (PDAC) (A) Gross pancreatic histology of CTR and KO KPC and KFC mice with PDAC. Norm: normal, G: glandular, MD: moderately differentiated, U: undifferentiated, C:cystic, 1°: greater or equal to 50%

of pancreas, 2°: >25% but <50%. (B) CK-19 staining of lung tissues from *Tigar* KO KPC and KFC. * = PDAC metastasis in lung. Scale bar, 100µm. (C) Numbers of organs (Lv, liver; Ln, lymph node; Sp, spleen; P, peritoneal) of CTR and KO KPC animals with or without metastasis. Data analysed by Fisher exact test. (D) PDAC-free survival of KPC animals (expressing R270H mutant p53) with (Nrf2$^{+/+}$, n = 21) and without NRF2 (Nrf2$^{-/-}$, n = 13). Data were analyzed by log rank test.

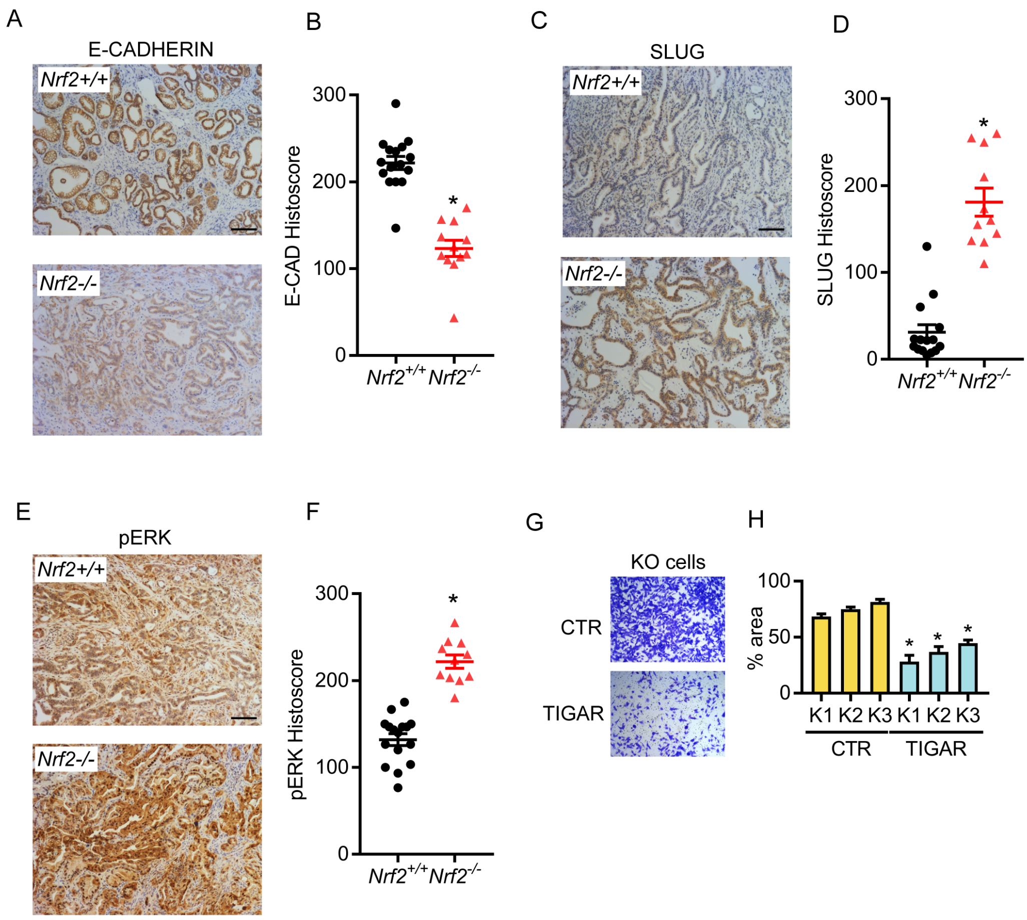

Figure S3. Related to Figure 3. NRF2 loss triggers a ERK-EMT signalling cascade in PDAC, similar to loss of TIGAR. Addition of recombinant TIGAR can decrease the invasiveness of *Tigar* KO PDAC (A and B) E-CADHERIN staining (A) and quantification (B) of $Nrf2^{+/+}$ and $Nrf2^{-/-}$ KPC tumours. * $p<0.05$ compared to $Nrf2^{+/+}$. (C and D) SLUG staining (C) and quantification (D) of $Nrf2^{+/+}$ and $Nrf2^{-/-}$ KPC tumours. * $p<0.05$ compared to $Nrf2^{+/+}$. (E and F) pERK staining (E) and quantification (F) of $Nrf2^{+/+}$ and $Nrf2^{-/-}$ KPC tumours. * $p<0.05$ compared to $Nrf2^{+/+}$. (G and H) Representative images (G) and quantification (H) of transwell migration assay of *Tigar* KO PDAC cells without (CTR) or with (TIGAR) recombinant TIGAR. * $p<0.05$ compared to CTR K1-3. Error bars represent mean ± SEM. (B,D,F) data analysed by two-tailed Student's *t* test, and (H) one way ANOVA with Tukey post hoc test. Scale bar, 100µm.

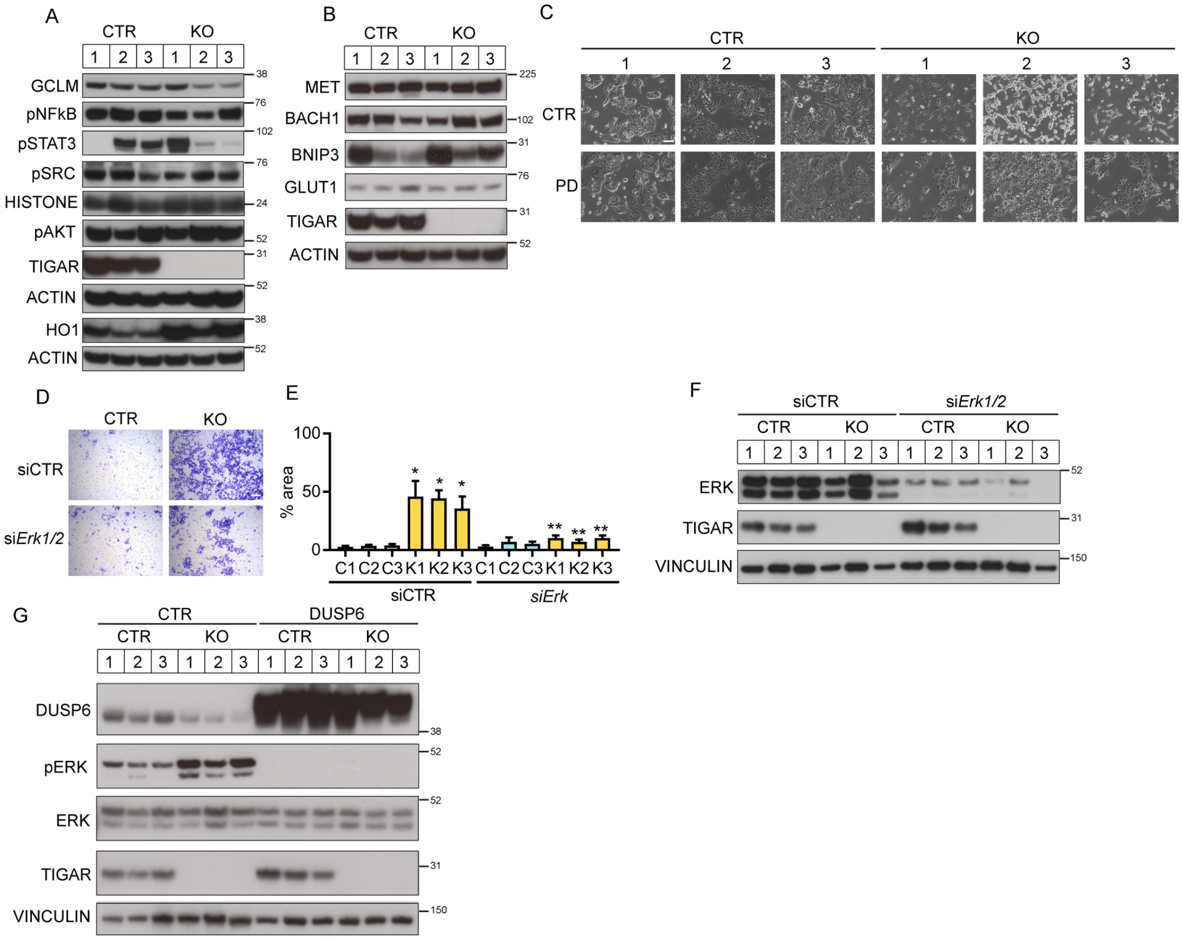

Figure S4. Related to Figure 4. Effect of TIGAR on ROS signalling to drive a mesenchymal phenotype via ERK pathway (A and B) Western blot analysis of CTR and TIGAR KO PDAC KFC cells. (A) GCLM, NFkB, pSTAT3, pSRC with the loading control HISTONE were detected on one blot; pAKT, TIGAR with the loading control ACTIN were detected on a second parallel blot; HO1 with loading control ACTIN (bottom) were detected on a third parallel blot. (B) MET, BACH1, BNIP3, GLUT1, TIGAR with the loading control ACTIN were detected on one blot. (C) Photomicrograph of CTR and TIGAR KO PDAC KFC cells treated without (CTR, vehicle treated) or with PD98059 (PD, 50µM). (D and E) Representative images (D) and quantification (E) of transwell migration assay of CTR and TIGAR KO KFC PDAC cells after knockdown of *Erk*. * $p<0.05$ K1-3 compared to C1-3, ** $p<0.05$ K1-3 with si*Erk* compared to K1-3 with siCTR. (F) Verification of *Erk* knockdown by Western blot analysis. ERK, TIGAR and the loading control VINCULIN were detected on one blot. (G) Western blot analysis of CTR and TIGAR KO KFC PDAC cells after expression of DUSP6. DUSP6, pERK, ERK, TIGAR and the loading control VINCULIN were detected on one blot. (E) n=3 independent experiments for each cell lines. Error bars represent mean ± SEM, data analysed by one way ANOVA with Tukey post hoc test. Scale bar, 100µm

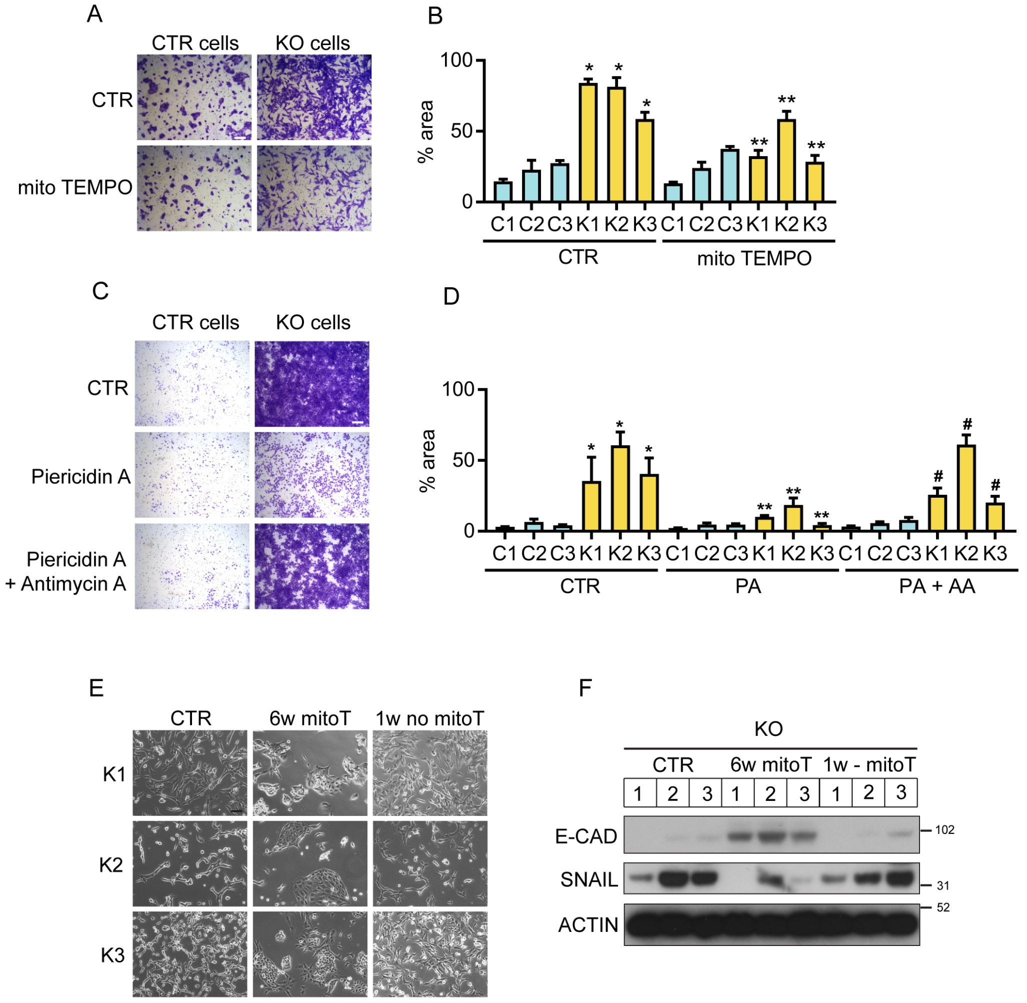

Figure S5: Related to Figure 5. *Tigar* deficiency induced migration can be reduced by limiting mitochondrial ROS. Representative images (A) and quantification (B) of transwell migration assay of CTR and TIGAR KO KFC PDAC cells with mito-TEMPO (100µM) or without (CTR, vehicle). * *p<0.05* K1-3 compared to C1-3, ** *p<0.05* mito-TEMPO treated K1-3 compared to CTR treated K1-3. (C and D) Representative images (C) and quantification (D) of transwell migration assay of CTR and KO PDAC cells with Piericidin-A (PA, 1µM), or combination of Piericidin and Antimycin A (AA, 1µM), or without (CTR, vehicle). * *p<0.05* K1-3 compared to C1-3, ** *p<0.05* PA treated K1-3 compared to CTR treated K1-3, # *p<0.05* PA+AA treated K1-3 compared to PA treated K1-3. (E) Representative images of KO KFC PDAC cells at indicated timepoints continuously treated with mito-TEMPO (mitoT) (50µM) and subsequent removal of mito-TEMPO for one week (1w no mitoT). (F) Representative Western blot analysis of one set of the samples in (E). E-CAD, SNAIL and the loading control ACTIN were detected on one blot. w= week. n=3 independent experiments for each cell lines. (B,D) Error bars represent mean ± SEM, and data analysed by one way ANOVA with Tukey post hoc analysis. Scale bar, 100µm.

A

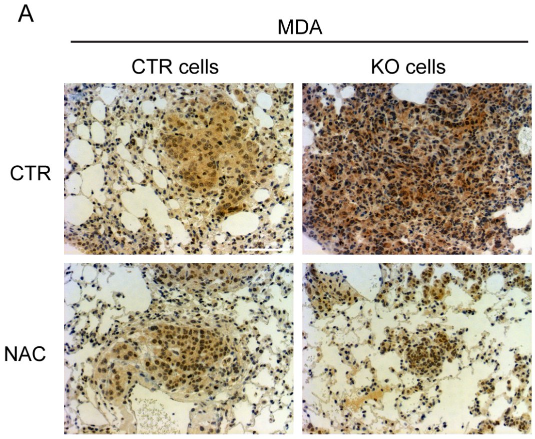

Figure S6. Related to Figure 6. MDA staining in TIGAR deficient metastases. (A) MDA staining of lung tissues from NAC treated or CTR (normal drinking water) treated animals tail vein injected with CTR and KO PDAC KFC cells. Scale bar, 100µm.
